# Orientation-Dependent Mechanical Response and Composite Action of Rectangular Pultruded GFRP Profiles and Integrated Sandwich Panels

**DOI:** 10.3390/ma19183906

**Published:** 2026-09-14

**Authors:** Juan Han, Hai Fang

**Affiliations:** 1 College of Urban Construction, Jiangsu Open University, Nanjing 210036, China; hanjuan_060101@163.com; 2 College of Civil Engineering, Nanjing Tech University, Nanjing 211816, China

**Keywords:** pultruded GFRP profile, integrated sandwich panel, profile orientation, composite action, flatwise compression

## Abstract

**Highlights:**

- V orientation increased bending peak loads by up to 26.06% for individual profiles.
- V-oriented panels gained 22.74% in bending but lost 28.24% in compression.
- Integrated panels exceeded component-wise baselines by up to 78.44%.
- Profile orientation should be selected according to the governing load state.
- Transverse web stability governs the design of pultruded-profile cores.
- Integrated fabrication promotes load redistribution and post-damage resistance.

**Abstract:**

Pultruded glass fibre-reinforced polymer (GFRP) profiles are promising structural cores for lightweight sandwich panels, but their strong longitudinal response is accompanied by weak transverse resistance and local web instability. A matched experimental programme examined rectangular hollow pultruded GFRP profiles and vacuum-infused sandwich panels under four-point bending, short-span three-point bending and flatwise compression in H and V orientations. Changing from H to V increased the peak loads of individual profiles by 9.35% in four-point bending and by 26.06% in short-span loading but reduced the flatwise compressive peak load by 30.12%. For integrated panels, the same reorientation increased the four-point bending peak load by 22.74%, caused only a marginal change in short-span capacity and reduced the flatwise compressive peak load by 28.24%. Failure involved local web buckling, web shear damage, longitudinal splitting, laminate delamination and interfacial debonding. Continuous face sheets and wrapping laminates restrained local deformation and contributed to substantial post-damage resistance, consistent with load redistribution within the integrated section. Relative to a baseline comprising three individual profiles and the corresponding hollow FRP reference panel, integrated-panel capacities increased by 15.76%, 37.71% and 78.44% under four-point bending, short-span loading and flatwise compression, respectively. These results identify profile orientation and transverse web stability as key design parameters for pultruded-profile-core sandwich panels.

## 1. Introduction

Fibre-reinforced polymer (FRP) composites combine low density, corrosion resistance and high specific mechanical properties, making them suitable for lightweight load-bearing construction. In sandwich panels, separated face sheets resist bending while the core transfers shear and stabilises the faces; consequently, structural efficiency depends not only on the constituent materials but also on interface integrity and the continuity of load transfer between them [1].

Conventional sandwich cores include polymeric foams, honeycombs and natural materials such as balsa. Sharaf and Fam [2] showed through large-scale transverse tests that the response of GFRP-faced cladding panels was strongly influenced by core and interface behaviour. Dawood et al. [3] further demonstrated under static and fatigue bending that through-thickness insertions altered core shear and face–core interaction, whereas Mathieson and Fam [4] identified soft-core shear as the governing failure mechanism in GFRP sandwich panels. Lattice-reinforced foam, PET foam and balsa-core systems can improve stiffness or weight efficiency, but core shear, local crushing, face wrinkling, interfacial debonding, creep and environmental sensitivity remain recurring limitations [5,6,7,8,9]. These vulnerabilities motivate structural cores that carry load directly rather than merely separating the face sheets.

Pultruded GFRP profiles are attractive structural cores because predominantly longitudinal fibres provide high axial and flexural efficiency, while hollow or multicellular geometries use material economically [10]. Keller and Schollmayer [11] found that interaction among pultruded deck units affected plate bending and deflection, and Xin et al. [12] combined experiments and analytical modelling to relate profile geometry to bridge-deck flexural response. Their longitudinal efficiency is nevertheless accompanied by comparatively weak transverse properties. Wu and Bai [13] and Chen and Wang [14] showed that concentrated bearing can trigger web crippling, web buckling and cracking near wall–web junctions; Al-Saadi et al. [15] confirmed that fibre orientation and lay-up strongly affect the mechanical properties of hollow pultruded profiles. Local-bearing tests by Xin et al. [16], transverse panel tests by Sá et al. [17], and combined experiments and finite-element analyses by Sun et al. [18] further demonstrated that loading position, shear span and section geometry modify local instability. Almeida-Fernandes et al. [19,20] quantified the effects of bearing length, lay-up and transverse fracture behaviour, while subsequent studies addressed non-uniform fibre–resin distributions, web perforations, geometry optimisation, built-up sections, local strengthening and stiffened wall–web junctions [21,22,23,24,25,26]. Reviews and recent experimental, numerical and design studies confirm that web slenderness, restraint and loading configuration govern transverse resistance [27,28,29,30]. Multicellular columns and full-scale footbridges also show the structural potential of assembled pultruded systems but retain sensitivity to local stability and connection details [31,32].

Composite action can be promoted by bonding, bolting, infill, external wrapping and vacuum infusion. Satasivam et al. [33] reported that adhesively bonded modular GFRP floor units could develop strong interaction, whereas Satasivam and Bai [34] found that bolted assemblies generally provided more limited continuity. Modular wall and square-hollow-section assemblies similarly showed that connections and panel restraint govern load sharing and failure progression [35,36]. For pultruded-profile-core sandwich panels, Shi et al. [37] linked short-span response to core shear and interfacial debonding, while Zhu et al. [38] showed that multiaxial wrapping modified flexural failure and post-peak resistance. CFRP strengthening [39] and multicavity-core configurations [40] further improved selected responses, yet local core damage and face-sheet failure remained closely coupled. Collectively, these studies indicate that continuous face sheets and wrapping laminates can restrain profile deformation, transfer load between adjacent profiles and preserve alternative load paths after local damage.

Despite these advances, direct comparisons between rectangular hollow pultruded GFRP profiles and sandwich panels manufactured from the same profile type remain limited. In particular, it remains unclear whether the effects of H and V orientations observed in individual profiles are retained after integration into sandwich panels and whether these effects are consistent across different loading conditions. A further question is how the measured panel peak load compares with a reference based on the capacities of separately tested profiles and the corresponding hollow FRP panel. Examining these questions together links the orientation-dependent response of individual profiles to the response and apparent capacity enhancement of the integrated panels.

The objective of this study is to evaluate how profile orientation affects the mechanical response of individual rectangular hollow pultruded GFRP profiles and integrated sandwich panels under four-point bending, short-span three-point bending and flatwise compression. Individual profiles and vacuum-infused panels containing three profiles, face sheets and wrapping laminates are tested in H and V orientations. Load–displacement response, strain development, observed damage and peak load are compared to examine whether orientation effects are consistent across loading conditions and between the two structural levels. For the H-oriented configuration, the panel peak load is also compared with a baseline calculated as three times the individual-profile peak load plus the peak load of the corresponding hollow FRP panel. These comparisons assess orientation-dependent behaviour and the apparent capacity enhancement associated with integrated fabrication.

## 2. Experimental Programme

### 2.1. Materials and Structural Configuration

#### 2.1.1. Rectangular Hollow Pultruded GFRP Profiles

Rectangular hollow pultruded GFRP profiles with outer dimensions of 50 mm × 80 mm and a nominal wall thickness of 4 mm were used. Continuous fibres were aligned predominantly with the profile axis. Two cross-sectional orientations were defined relative to the loading direction. In the H orientation, the profile had a width of 80 mm and a depth of 50 mm, whereas in the V orientation, the width was 50 mm, and the depth was 80 mm. The corresponding depth-to-width ratios were 0.625 and 1.60, respectively. The terms H and V are used throughout the manuscript to denote these two cross-sectional orientations. The 4 mm value represents the nominal wall thickness of the pultruded profiles; specimen-to-specimen wall-thickness variation was not systematically recorded.

#### 2.1.2. Integrated Sandwich Panels with Pultruded-Profile Cores

The face sheets and wrapping laminates were manufactured from G100 unsaturated polyester resin and biaxial woven glass fabrics with fibre orientations of 0°/90° and ±45°. The fabrics had an areal density of 800 g/m^2^, with an approximate cured ply thickness of 0.6 mm. Accordingly, the two-ply wrapping laminate and three-ply face sheets had nominal thickness values of approximately 1.2 and 1.8 mm, respectively.

**Figure 1:**
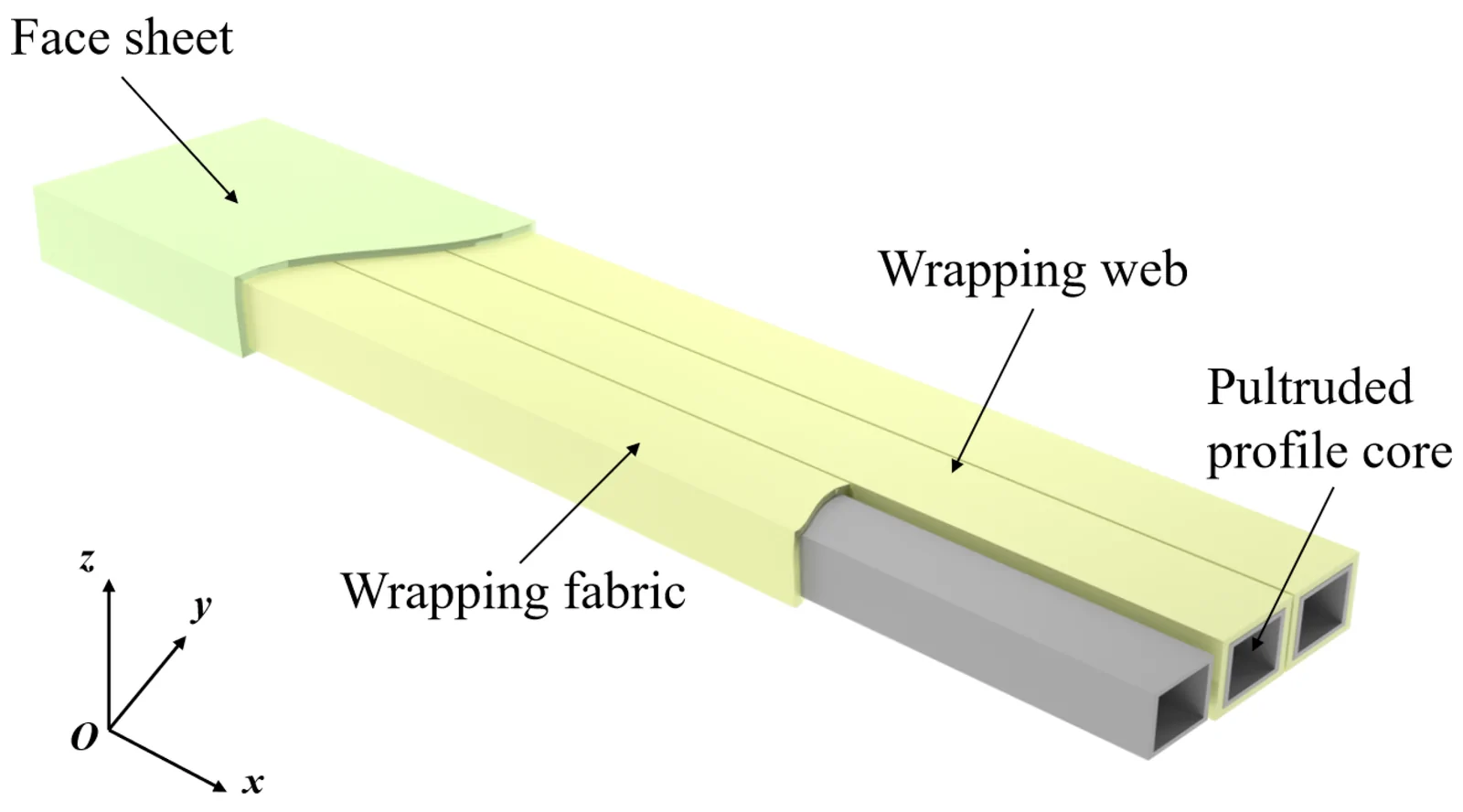
Schematic configuration of the integrated pultruded-profile-core sandwich panel.

As illustrated in Figure 1, each integrated sandwich panel comprised three rectangular hollow pultruded GFRP profiles and externally infused fibre-reinforced composite layers. The profiles were aligned side by side along the longitudinal direction to form the structural core. Continuous fabrics were wrapped around the profiles and combined with top and bottom face sheets; vacuum infusion subsequently consolidated the dry fabrics and the pultruded profiles into an integrated sandwich section.

The wrapping lay-up was [(±45°)_2_], and the top and bottom face sheets used [(0°,90°)/(±45°)/(0°,90°)] lay-ups. Panels were manufactured with either H- or V-oriented cores, giving nominal core depths of 50 and 80 mm, respectively. All other parameters, including profile geometry, wall thickness, number of profiles and external fabric lay-ups, were identical.

### 2.2. Specimen Design and Identification

#### 2.2.1. Specimen Identification

The designation identifies the loading condition, profile core, orientation and specimen scale. F, S and C denote four-point bending, short-span three-point bending and flatwise compression, respectively, while P denotes a pultruded profile. The letters H and V denote the two cross-sectional orientations defined in Section 2.1.1: 80 mm wide × 50 mm deep for H and 50 mm wide × 80 mm deep for V. The term “flatwise compression” refers to the loading condition rather than the H orientation, and both H- and V-oriented specimens were tested under this loading condition. The suffix “-S” identifies an individual-profile specimen; designations without this suffix refer to integrated panels containing three profiles. Thus, FPH-S is an H-oriented individual profile tested in four-point bending, whereas FPH is the corresponding integrated panel.

#### 2.2.2. Individual-Pultruded-Profile Specimens

Individual profiles were tested under four-point bending, short-span three-point bending and flatwise compression. Each loading condition included H- and V-oriented specimens, with three nominally identical replicates for each configuration (*n* = 3), giving 18 individual-profile specimens in total. The specimen matrix is given in Table 1.

The four-point bending specimens were 1000 mm long with an 800 mm span, and the short-span three-point bending specimens were 400 mm long with a 300 mm span. Flatwise compression specimens were cut to a longitudinal length of 100 mm. Selected specimens were used only to illustrate the characteristic load–displacement, load–strain and damage responses observed within each three-specimen group. The plotted specimen was not treated as a statistical substitute for the replicate tests. For the FPH-S and SPH-S strain responses, the third specimen was used for illustration because the three specimens showed similar response trends.

#### 2.2.3. Integrated Sandwich-Panel Specimens

Integrated panels were tested under the same three loading conditions and in both core orientations. One specimen was tested for each integrated-panel configuration (*n* = 1), giving six integrated-panel specimens in total. Specimen lengths and spans matched those of the corresponding individual-profile tests: 1000 mm with an 800 mm span for four-point bending, 400 mm with a 300 mm span for short-span three-point bending, and 100 mm longitudinal length for flatwise compression. Table 2 summarises the configurations.

One hollow FRP reference panel was tested for each loading condition (FH, SH and CH; *n* = 1 for each configuration), giving three reference panels in total. These specimens were used for the component-wise capacity comparison and had the same nominal external fabric lay-ups and overall dimensions as the corresponding H-oriented integrated panels. The actual resin uptake of individual panels was not separately measured.

### 2.3. Specimen Fabrication

Individual specimens were cut from rectangular hollow pultruded GFRP profiles of the same production specification. Cut edges were finished carefully to minimise machining damage within the test regions.

**Figure 2:**
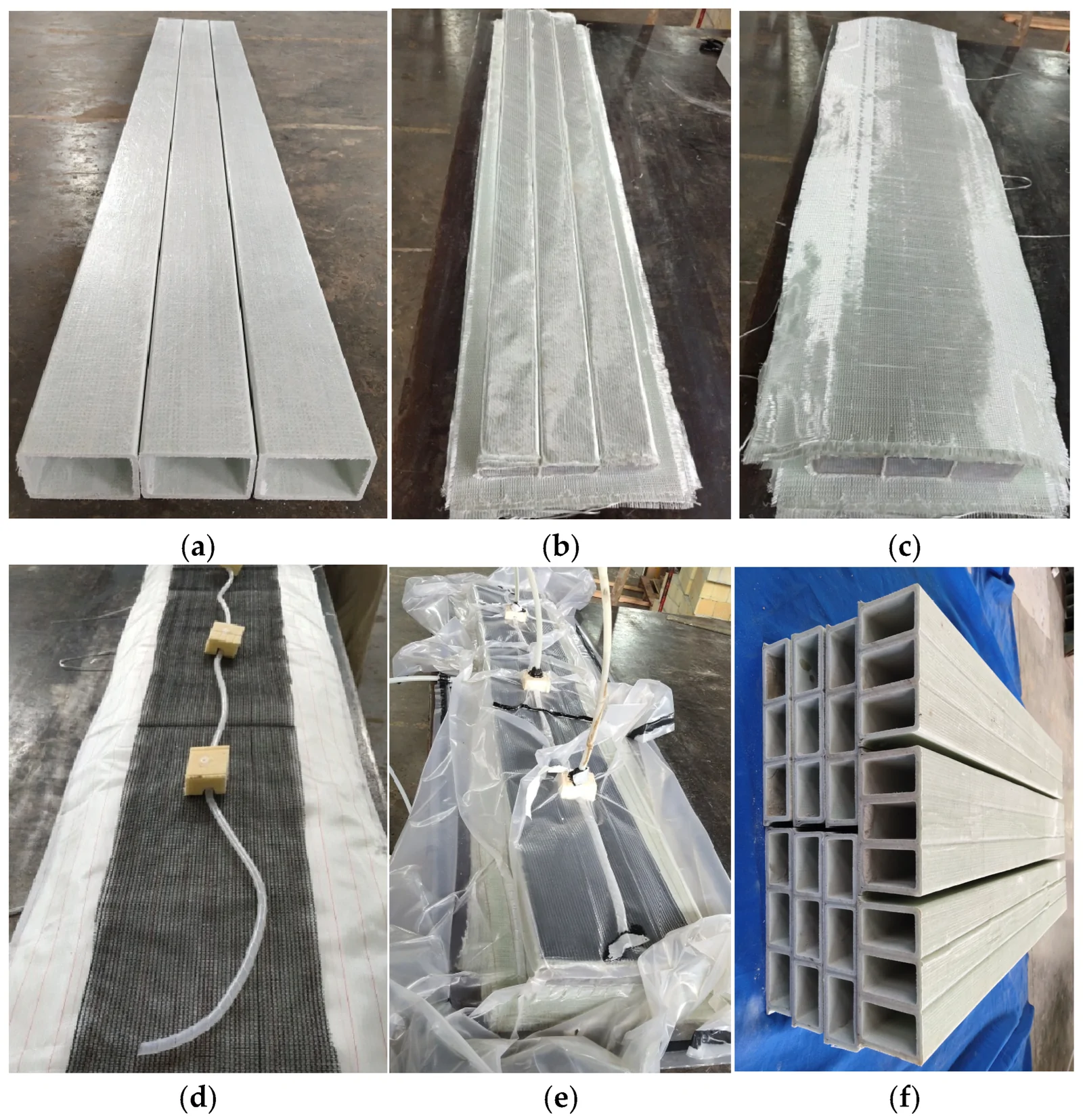
Fabrication procedure for the integrated pultruded-profile-core sandwich panels: (**a**) pultruded profiles; (**b**) placement of wrapped profiles on the bottom face-sheet fabrics; (**c**) top face-sheet lay-up; (**d**) arrangement of peel ply, flow media and infusion tubing; (**e**) vacuum infusion; and (**f**) fabricated panels.

The integrated panels were manufactured by vacuum infusion, as illustrated in Figure 2. The pultruded profiles were first cut to the required length, and their ends were sealed. Each profile was then wrapped with two plies of ±45° glass fabric. Three wrapped profiles were arranged side by side in the prescribed H or V orientation on the bottom face-sheet fabrics, after which the top face-sheet fabrics were laid. Peel ply, flow media and other vacuum-infusion consumables were then installed. The assembly was sealed with a vacuum bag and evacuated before resin infusion. After curing, the panels were demoulded, trimmed and cut to the dimensions required for the corresponding mechanical tests. The H- and V-oriented integrated panels were fabricated in the same vacuum-infusion batch using the same constituent materials, fabric lay-ups and processing conditions.

### 2.4. Test Setup and Instrumentation

#### 2.4.1. Four-Point Bending Tests

**Figure 3:**
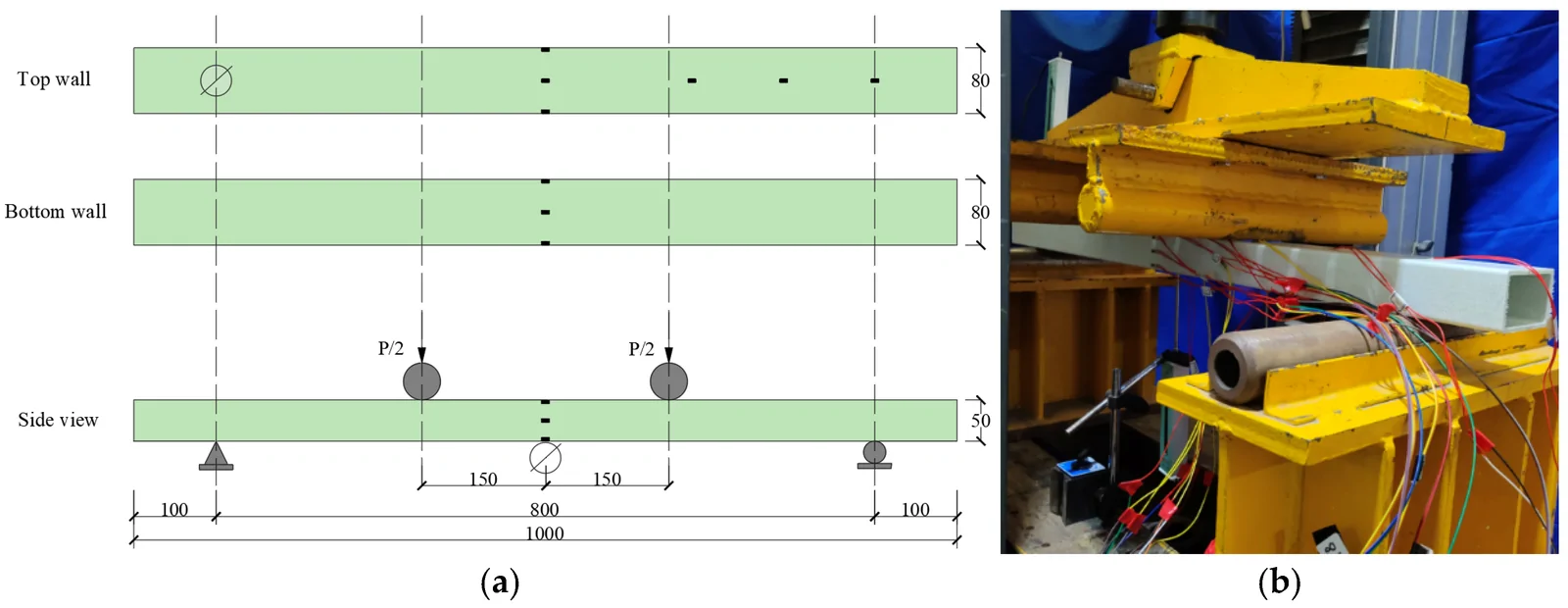
Four-point bending test setup and instrumentation for individual-pultruded-profile specimens. (**a**) Loading and instrumentation scheme. (**b**) Experimental setup. Arrows indicate the loading direction, and dashed lines indicate the corresponding loading and support positions.

Four-point bending tests on the individual profiles were conducted in displacement control using an MTS electro-hydraulic servo-controlled universal testing machine (MTS Systems Corporation, Eden Prairie, MN, USA) with a load capacity of 200 kN. The loading rate was 2 mm/min, and the span was 800 mm. The loading and instrumentation scheme and the test setup are shown in Figure 3a and Figure 3b, respectively.

Midspan deflection was measured using a vertical displacement transducer with a measurement range of 100 mm. Electrical resistance strain gauges were bonded to the top and bottom walls to record longitudinal compressive and tensile strains, respectively, and additional strain gauges were distributed through the depth of the midspan web to characterise the sectional strain distribution. Load, displacement and strain were acquired synchronously.

Integrated panels were tested over the same 800 mm span. A vertical displacement transducer measured midspan deflection, and longitudinal strain gauges were installed on the top and bottom face sheets and at several positions through the panel depth to characterise global response and sectional strain development.

#### 2.4.2. Short-Span Three-Point Bending Tests

**Figure 4:**
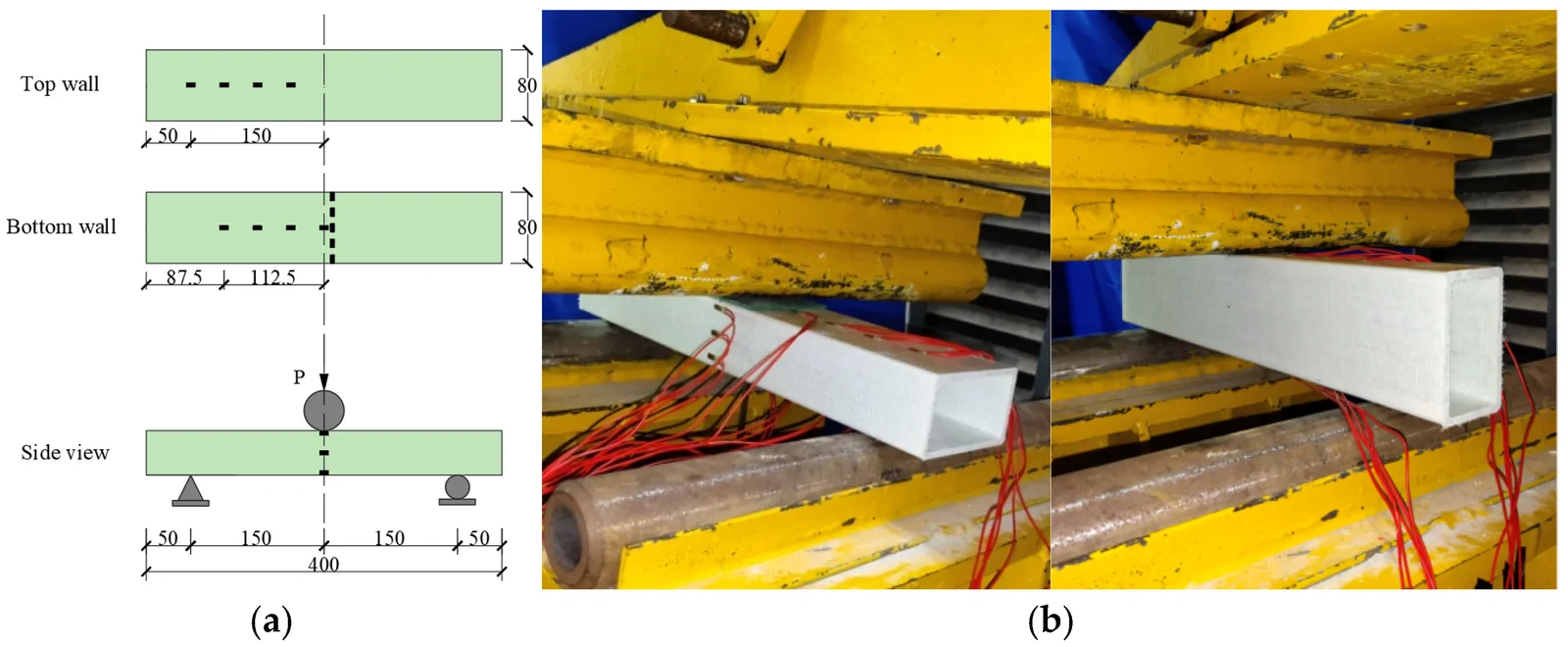
Short-span three-point bending test setup and instrumentation for individual-pultruded-profile specimens. (**a**) Loading and instrumentation scheme. (**b**) Experimental setup. Arrows indicate the loading direction, and dashed lines indicate the corresponding loading and support positions.

Short-span three-point bending tests on individual profiles were performed in displacement control on the same 200 kN testing machine. The loading rate was 2 mm/min, and the span was 300 mm. The loading and instrumentation scheme and the test setup are shown in Figure 4a and Figure 4b, respectively.

The low span-to-depth ratios increased the relative contributions of web shear deformation and local bearing beneath the loading point. The measured response was nevertheless produced by combined bending, shear and local bearing rather than pure shear. The configuration is therefore described throughout as short-span three-point bending, with its shear-dominated features discussed in Section 3.

**Figure 5:**
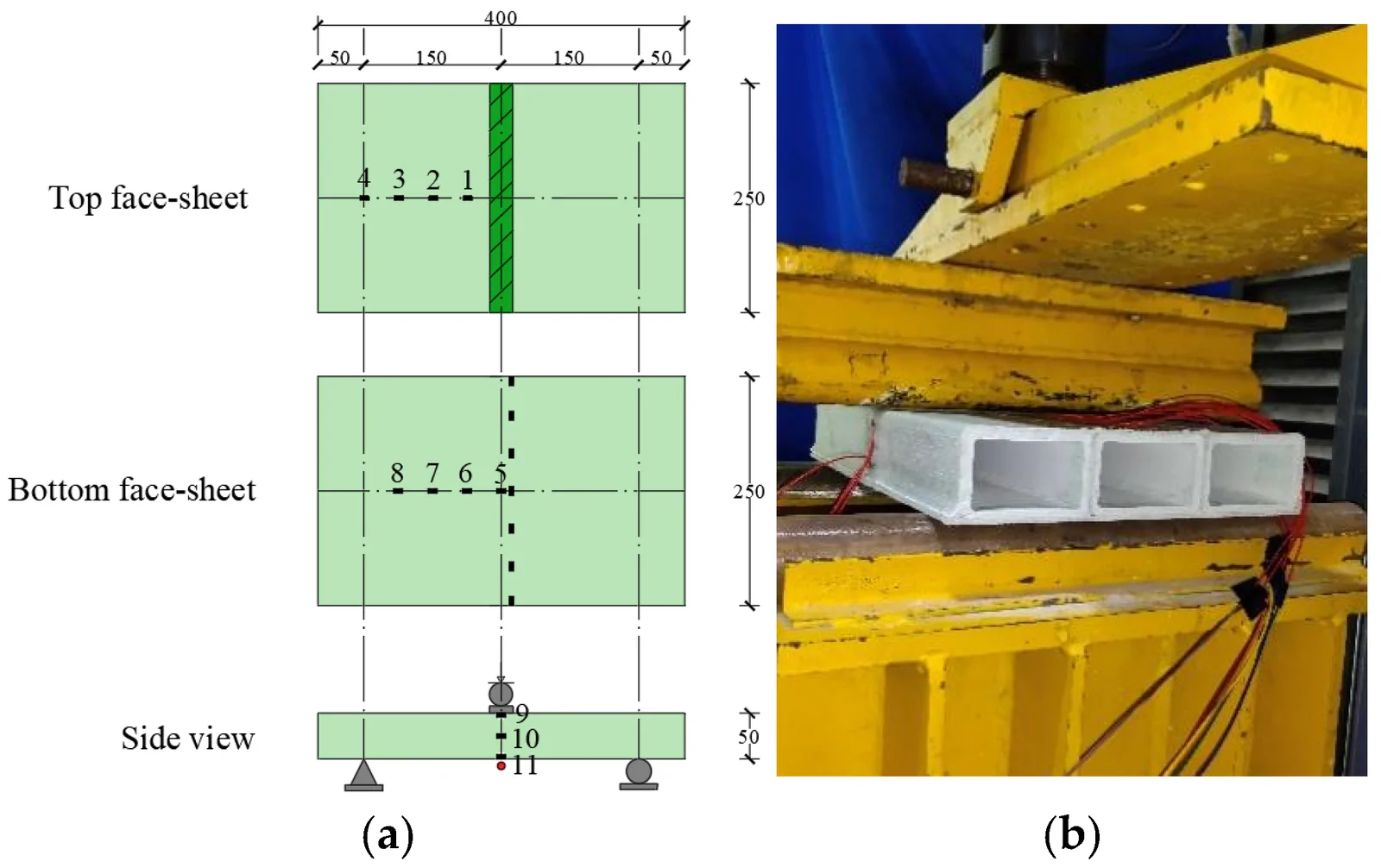
Short-span three-point bending test setup and instrumentation for integrated sandwich panels. (**a**) Loading and instrumentation scheme. (**b**) Experimental setup. The dashed line indicates the loading position at midspan.

Integrated panels were tested over the same 300 mm span with reference to ASTM C393 [41]. The loading and instrumentation arrangement is shown in Figure 5. Midspan displacement was recorded, and observations focused on local face-sheet indentation, damage to the internal profile webs and debonding between the pultruded profiles and surrounding wrapping laminates.

#### 2.4.3. Flatwise Compression Tests

Flatwise compression tests on the individual profiles and integrated panels were conducted on the 200 kN testing machine with reference to the loading procedure in ASTM C365 [42]. All specimens were 100 mm long in the profile direction. Each specimen was centred between two rigid platens, with full contact between the loaded surfaces and the platens and alignment of the specimen centre with the loading axis.

Loading was applied in displacement control at 2 mm/min until a pronounced loss of capacity or substantial damage occurred. Compressive load and crosshead displacement were recorded synchronously. The crosshead displacement was used directly without a separate correction for machine compliance.

For individual profiles, the recorded damage included outward web deformation, cracking at corners or wall surfaces, delamination within the profile walls and through-thickness cracking. For integrated panels, observations focused on the deformation and cracking of the profile webs and wrapping laminates, together with debonding at the profile–wrapping interfaces. Both H- and V-oriented configurations were tested to quantify the effect of profile orientation on flatwise compressive response.

### 2.5. Performance Evaluation Metrics

The peak load of each specimen, *P*_u_, was defined as the maximum value on the load–displacement curve, and *δ*_u_ was the corresponding displacement. When a specimen sustained initial damage, recovered capacity and subsequently reached a higher load, the initial damage load and final peak load were reported separately.

The flatwise compressive strength of an individual profile, *f*_c_, was calculated as

(1) $$f_{c}=\frac{P_{\max}}{A}$$

where *P*_max_ is the maximum compressive load and *A* is the nominal net cross-sectional area of the hollow profile, calculated as 976 mm^2^ from the nominal section dimensions.

The flatwise compressive chord modulus, *E*_c_, was determined from

(2) $$E_{c}=\frac{t\left(P_{0.003}-P_{0.001}\right)}{A\left(\delta_{0.003}-\delta_{0.001}\right)}$$

where *P*_0.001_ and *P*_0.003_ are the loads corresponding to compressive strains of 0.001 and 0.003, respectively; *δ*_0.001_ and *δ*_0.003_ are the associated displacements; and *t* is the initial specimen dimension in the loading direction. The chord modulus was calculated from the recorded crosshead displacement without machine-compliance correction.

The percentage change in peak load produced by changing from the H to the V orientation was calculated as

(3) $$\eta_{V/H}=\frac{P_{u,V}-P_{u,H}}{P_{u,H}}\times 100\%$$

where *P*_u,H_ and *P*_u,V_ are the peak loads of the H- and V-oriented specimens, respectively. Positive *η*_V/H_ denotes an increase for the V orientation, whereas a negative value denotes a reduction.

A component-wise superposition baseline was defined to separate the independently measured component capacities from the additional capacity observed in the integrated panel:

(4) $$P_{\mathrm{sum}}\;=\;3P_{u,\mathrm{profile}}\;+\;P_{u,\mathrm{cavity}}$$

The apparent capacity enhancement of the integrated panel relative to this baseline was calculated as

(5) $$\eta_{c}=\frac{P_{u,\mathrm{panel}}-P_{\mathrm{sum}}}{P_{\mathrm{sum}}}\times 100\%$$

where *P*_u,profile_ is the peak load of the individual profile, *P*_u,cavity_ is the peak load of the corresponding hollow FRP reference panel, and *P*_u,panel_ is the measured peak load of the integrated panel. The resulting index expresses the percentage by which the measured panel capacity exceeds the sum of the independently tested component capacities; it is used here as an experimental indicator of composite action rather than as an intrinsic material property.

## 3. Results and Discussion

### 3.1. Mechanical Response of Individual Rectangular Hollow Pultruded Profiles

#### 3.1.1. Four-Point Bending Response and Failure Mechanisms

**Figure 6:**
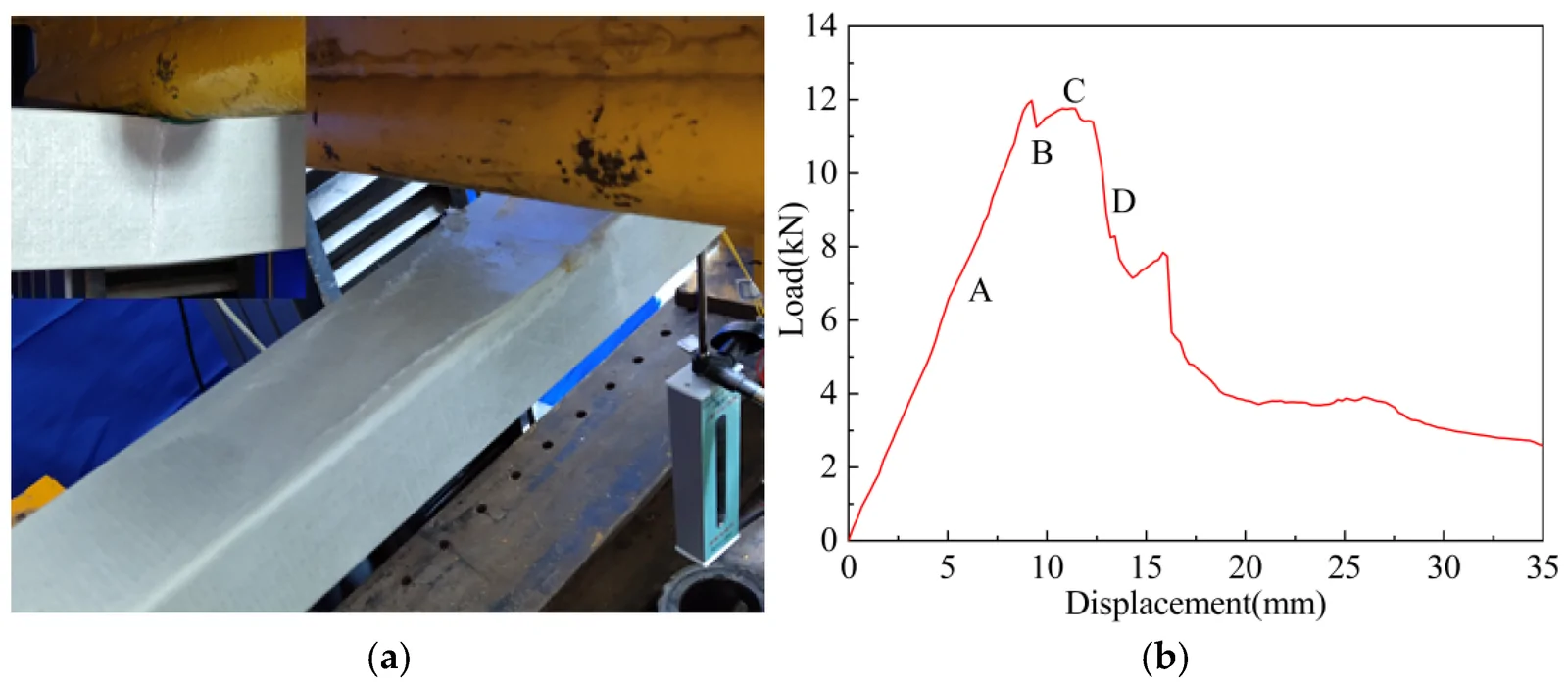
Failure mode and load–displacement response of a representative FPH-S specimen. (**a**) Failure mode. (**b**) Load–displacement response. Labels A–D denote the initial linear stage, local web buckling and cracking, short post-damage load recovery, and longitudinal splitting with rapid capacity loss, respectively.

Figure 6 presents the failure mode and load–displacement response of an illustrative FPH-S specimen. The labelled points A–D in Figure 6b denote characteristic stages of the observed response: A, the initial linear stage; B, local web buckling and cracking with a small load reduction; C, a short post-damage load-recovery stage; and D, longitudinal splitting at the wall–web junction followed by rapid capacity loss. The initial response was approximately linear, and no visible damage was observed. Local buckling then developed in the webs adjacent to the loading points, accompanied by vertical cracks extending through the web depth and a small load reduction. A brief plateau followed, but the recovered load remained below the preceding peak. Longitudinal splitting subsequently initiated at the upper wall–web junction near a loading point and propagated along the profile axis, producing a rapid loss of capacity. The FPH-S group had a mean peak load of 11.98 kN.

Damage was concentrated beneath the loading points rather than in the tensile region at midspan. The failure sequence therefore suggests control by the combined effects of local bearing, web instability and limited transverse resistance at the wall–web junction. Predominantly longitudinal fibres provided an easy propagation path for splitting along the profile axis, consistent with the abrupt post-peak reduction.

**Figure 7:**
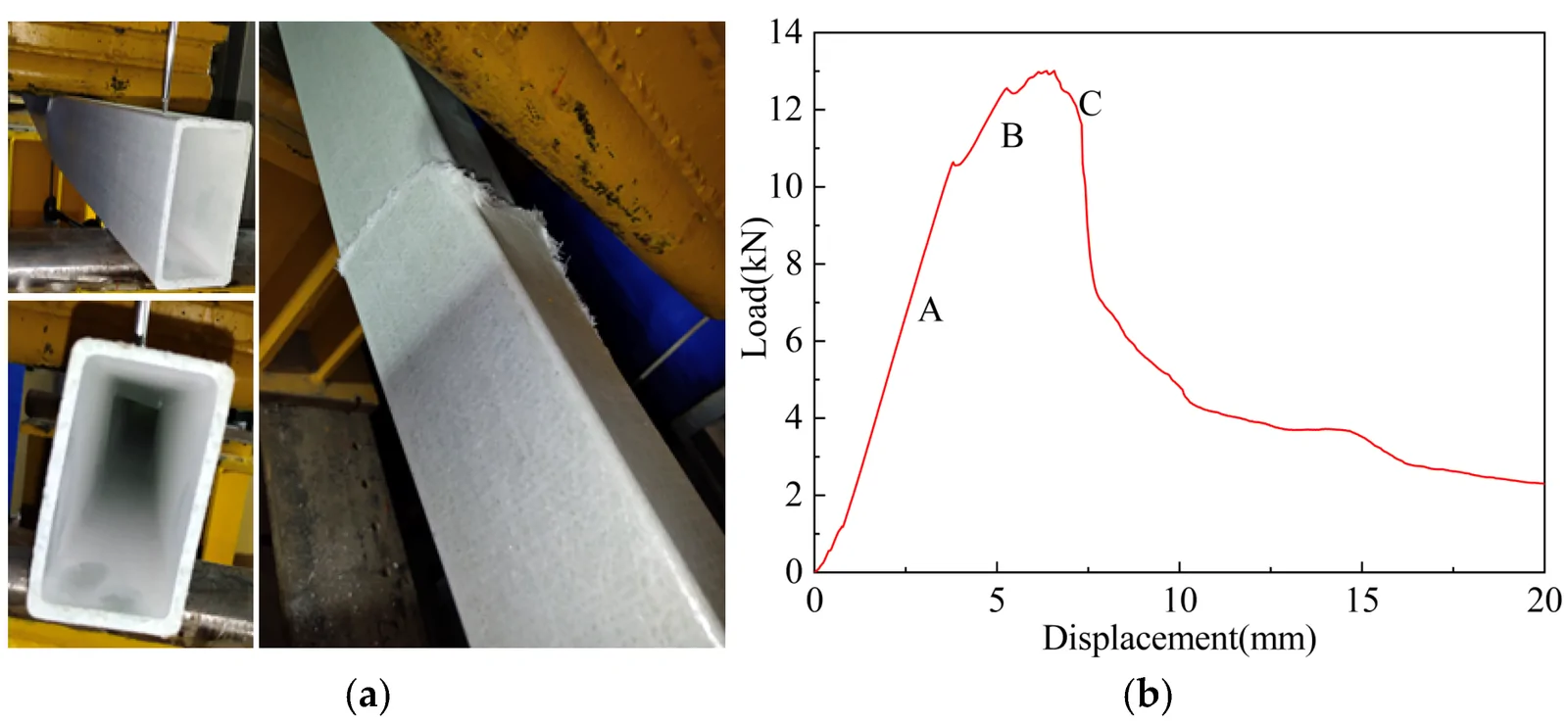
Failure mode and load–displacement response of a representative FPV-S specimen. (**a**) Failure mode. (**b**) Load–displacement response. Labels A–C denote the initial linear response, progressive local deformation and fibre fracture with stiffness degradation, and the peak-load stage associated with transverse cracking and longitudinal splitting, respectively.

As shown in Figure 7, the FPV-S specimen also showed an initially linear response. The webs near the loading points progressively deformed outwards, local fibre fracture developed and the tangent stiffness decreased. A transverse crack then formed around the loaded section and penetrated the profile wall, together with longitudinal splitting at the upper wall–web junction. Interaction among these damage modes caused the final rapid loss of capacity. The FPV-S group had a mean peak load of 13.10 kN.

#### 3.1.2. Flexural Strain Response

**Figure 8:**
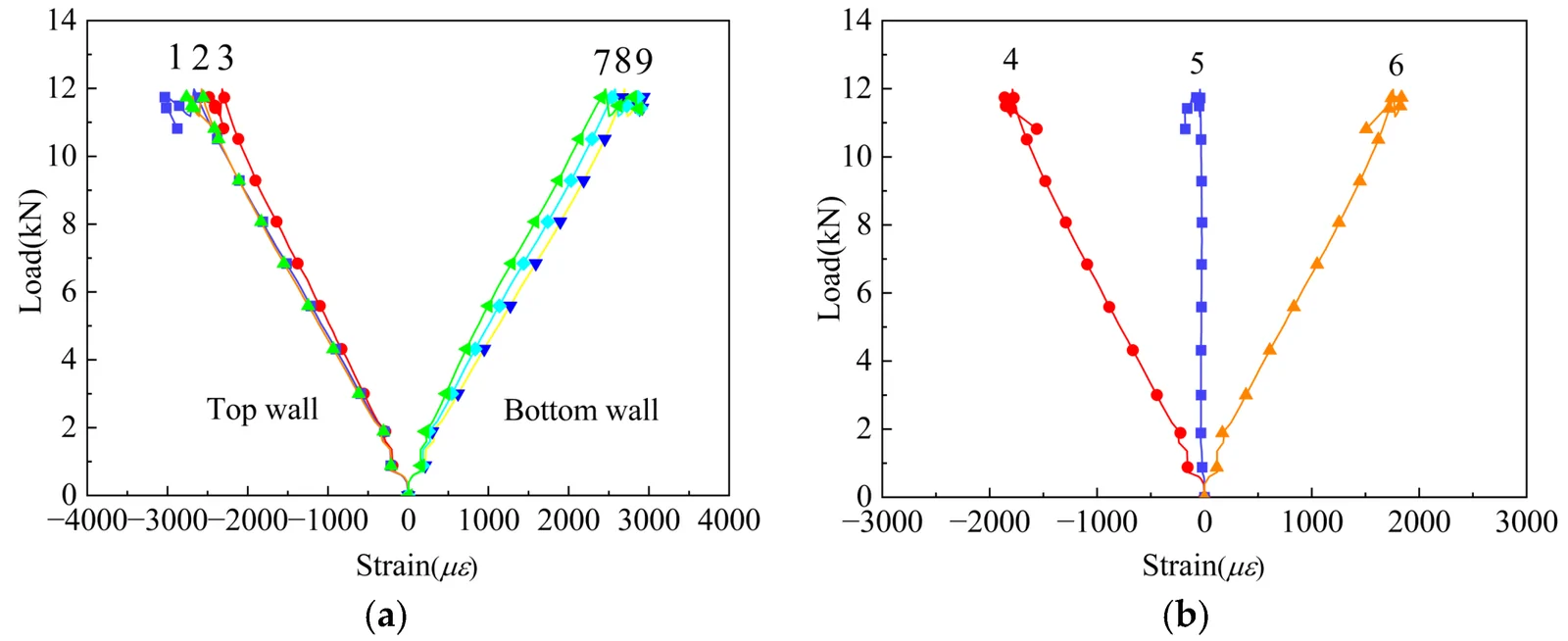
Load–strain responses of a representative FPH-S specimen. (**a**) Top- and bottom-wall responses. (**b**) Web responses. Numbers indicate the strain-gauge locations shown in the instrumentation scheme.

Figure 8 shows the load–strain response of a representative FPH-S specimen. Before peak load, longitudinal strains in the top and bottom walls varied approximately linearly with load. Local damage produced marked nonlinearity after the peak. At midspan, strain changed approximately linearly through the section depth; compressive strain in the upper wall and tensile strain in the lower wall were of similar magnitude, and the strain near mid-depth remained close to zero. The section therefore exhibited approximately plane sectional behaviour before substantial local damage developed.

The strain response was consistent with the observed failure location. A regular flexural strain distribution was retained at midspan up to peak load, whereas severe damage developed near the loading points. Peak capacity was therefore governed primarily by local bearing and web instability in the loaded regions rather than by attainment of a longitudinal material strain limit at midspan.

#### 3.1.3. Short-Span Three-Point Bending Response and Failure Mechanisms

**Figure 9:**
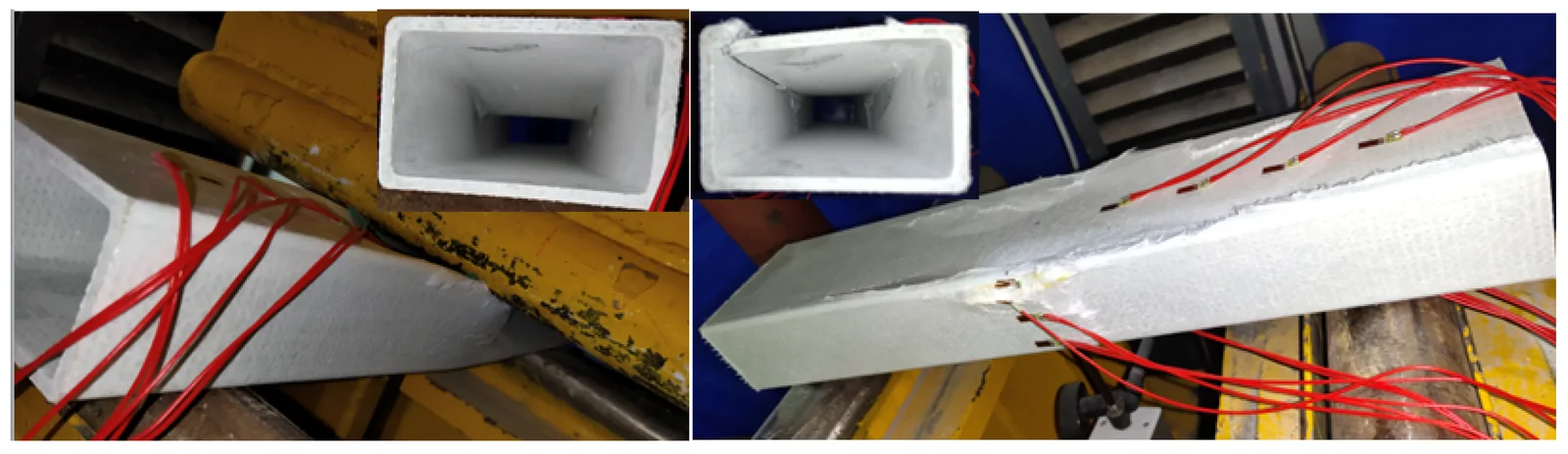
Failure modes of a representative SPH-S specimen.

**Figure 10:**
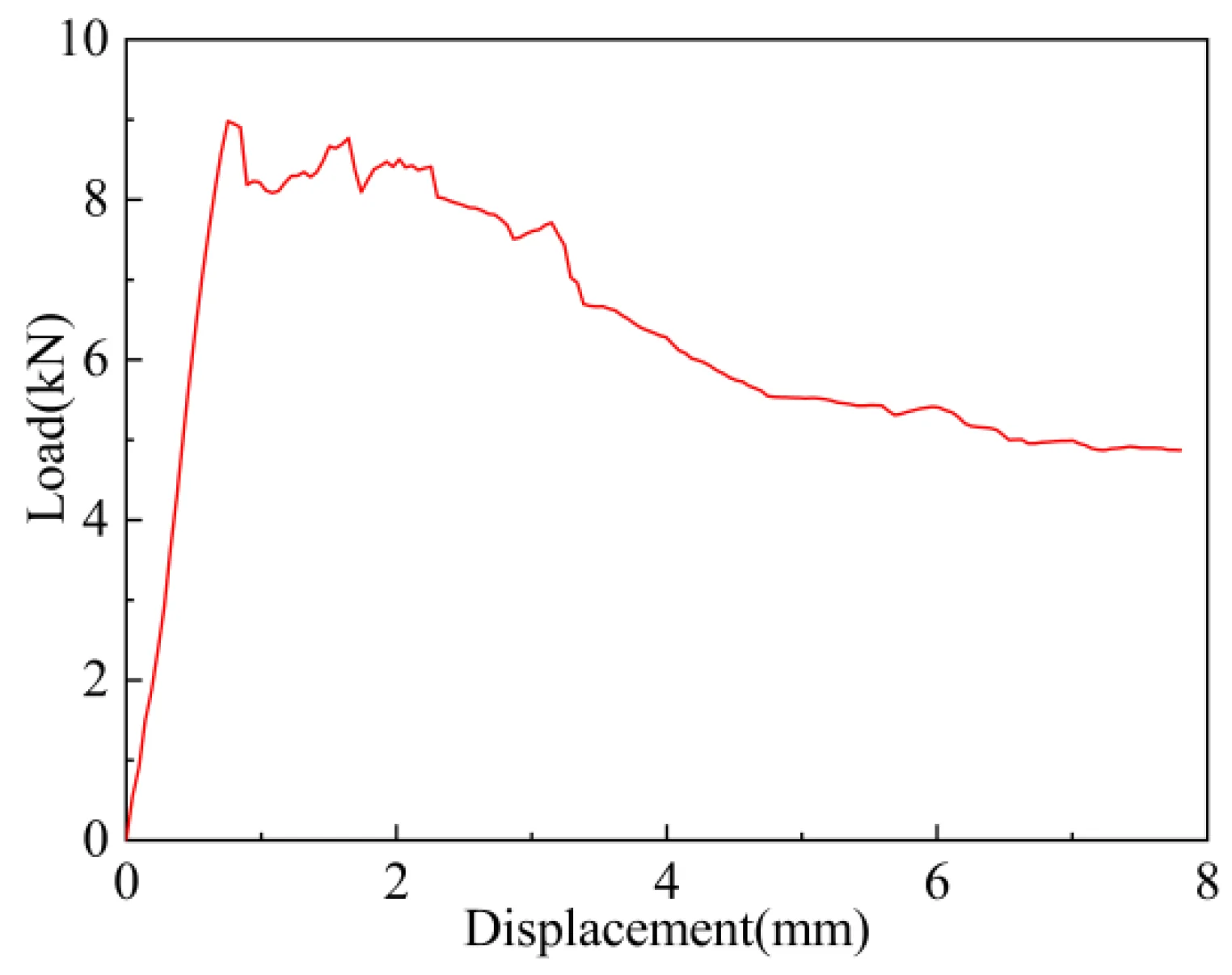
Load–displacement response of a representative SPH-S specimen.

Figure 9 and Figure 10 show the failure mode and load–displacement response of a representative SPH-S specimen, respectively. The response was approximately linear until successive fibre-fracture sounds were detected at 8.12 kN, marking the onset of local damage beneath the loading point. At the peak load of 8.98 kN, damage at the wall–web junction was accompanied by pronounced outward web deformation. The load then decreased to 8.08 kN.

The specimen recovered temporarily to 8.77 kN before longitudinal splitting developed near the loading point together with pronounced web shear damage. Cracks propagated towards the specimen end along the profile axis, leading to a gradual reduction in capacity. The sequence indicates interaction among local bearing, web shear damage and longitudinal splitting at the wall–web junction.

**Figure 11:**
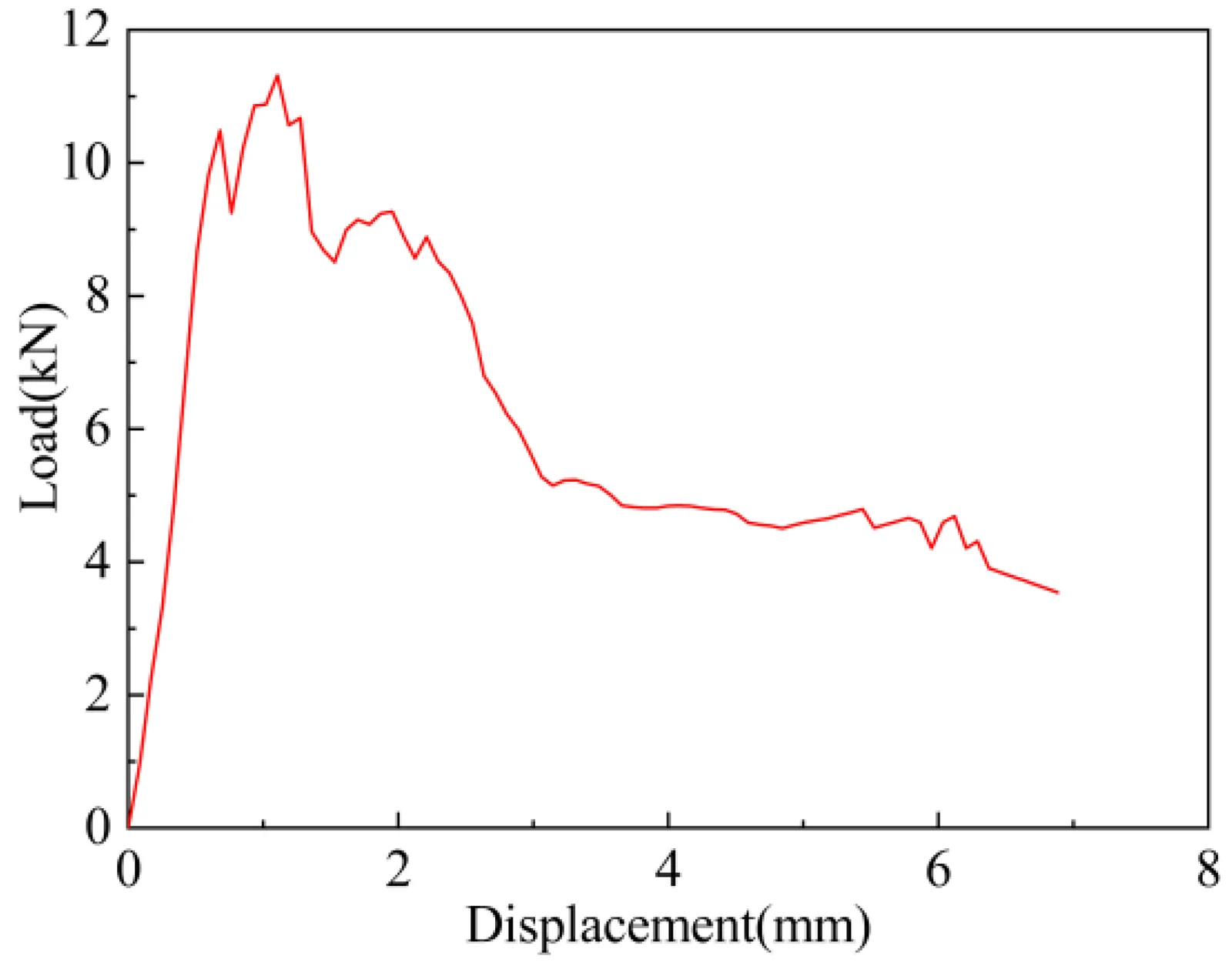
Load–displacement response of a representative SPV-S specimen.

**Figure 12:**
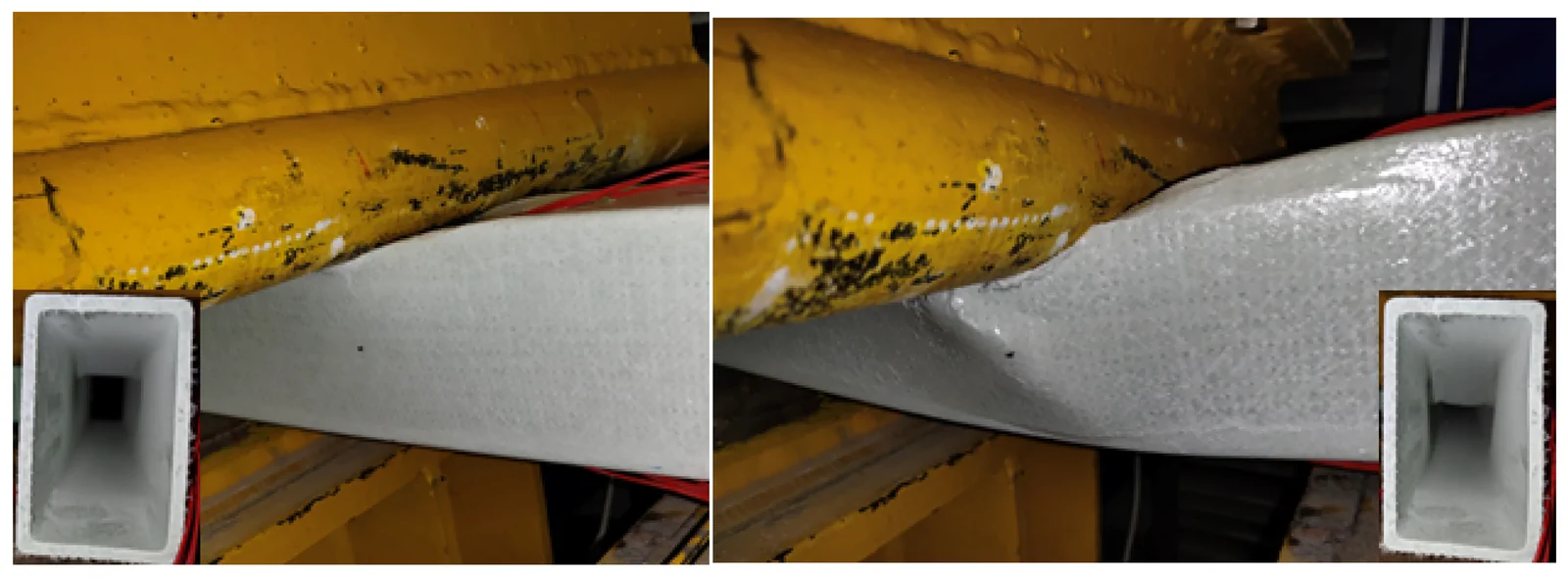
Failure modes of a representative SPV-S specimen.

In SPV-S, local buckling first appeared in the web beneath the loading point at 10.49 kN and reduced the load to 9.25 kN. Because this initial damage remained localised, the specimen recovered and reached a higher final peak of 11.32 kN. Longitudinal splitting at the wall–web junction and shear failure in the loaded web then reduced the load to 8.51 kN. Thereafter, capacity declined gradually and stabilised at approximately 4.85 kN. Figure 11 and Figure 12 show the corresponding load–displacement response and failure mode.

Accordingly, 10.49 kN was identified as the initial damage load and 11.32 kN as the final peak load. The final peak of SPV-S was 26.06% greater than that of SPH-S. The larger section depth appears to have increased the effective shear-resisting depth, but local web buckling and splitting at the wall–web junction continued to limit capacity.

#### 3.1.4. Strain Response Under Short-Span Three-Point Bending

**Figure 13:**
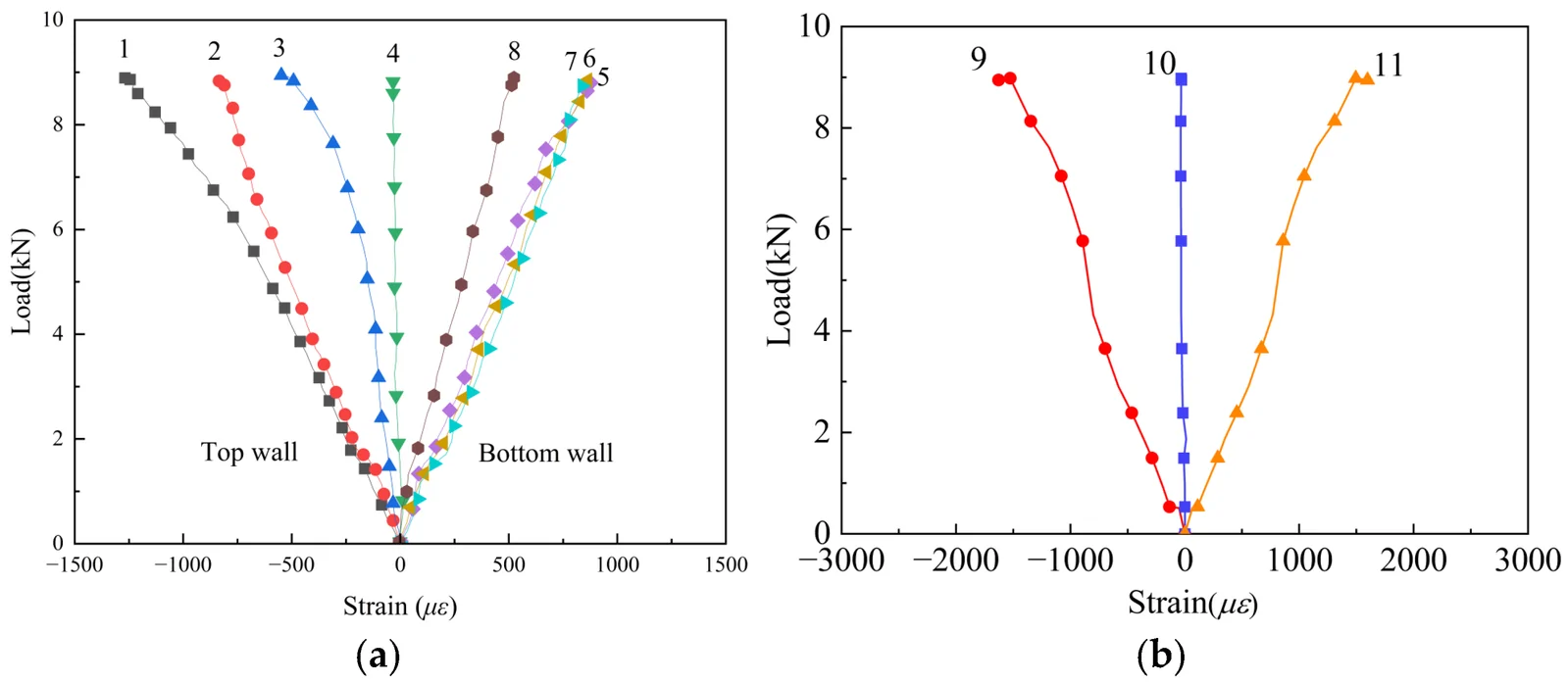
Load–strain responses of a representative SPH-S specimen. (**a**) Top- and bottom-wall responses. (**b**) Web responses. Numbers indicate the strain-gauge locations shown in the instrumentation scheme.

Figure 13 presents the load–strain response of a representative SPH-S specimen. Strains in the top and bottom walls varied approximately linearly with load before the peak. Subsequent web shear damage and cracking at the wall–web junctions produced pronounced nonlinearity. The maximum tensile strain in the bottom wall was approximately 0.16%, compared with a maximum compressive strain of approximately 0.09% in the top wall.

At midspan, the upper and lower measurement points were in compression and tension, respectively, while the strain near mid-depth remained close to zero. Thus, despite the increased influence of shear deformation and local bearing at the reduced span-to-depth ratio, appreciable global bending remained present at midspan. These results characterise the combined structural response to bending, shear and local bearing and should not be interpreted as a direct measurement of the material’s pure shear properties.

#### 3.1.5. Flatwise Compression Response and Failure Mechanisms

**Figure 14:**
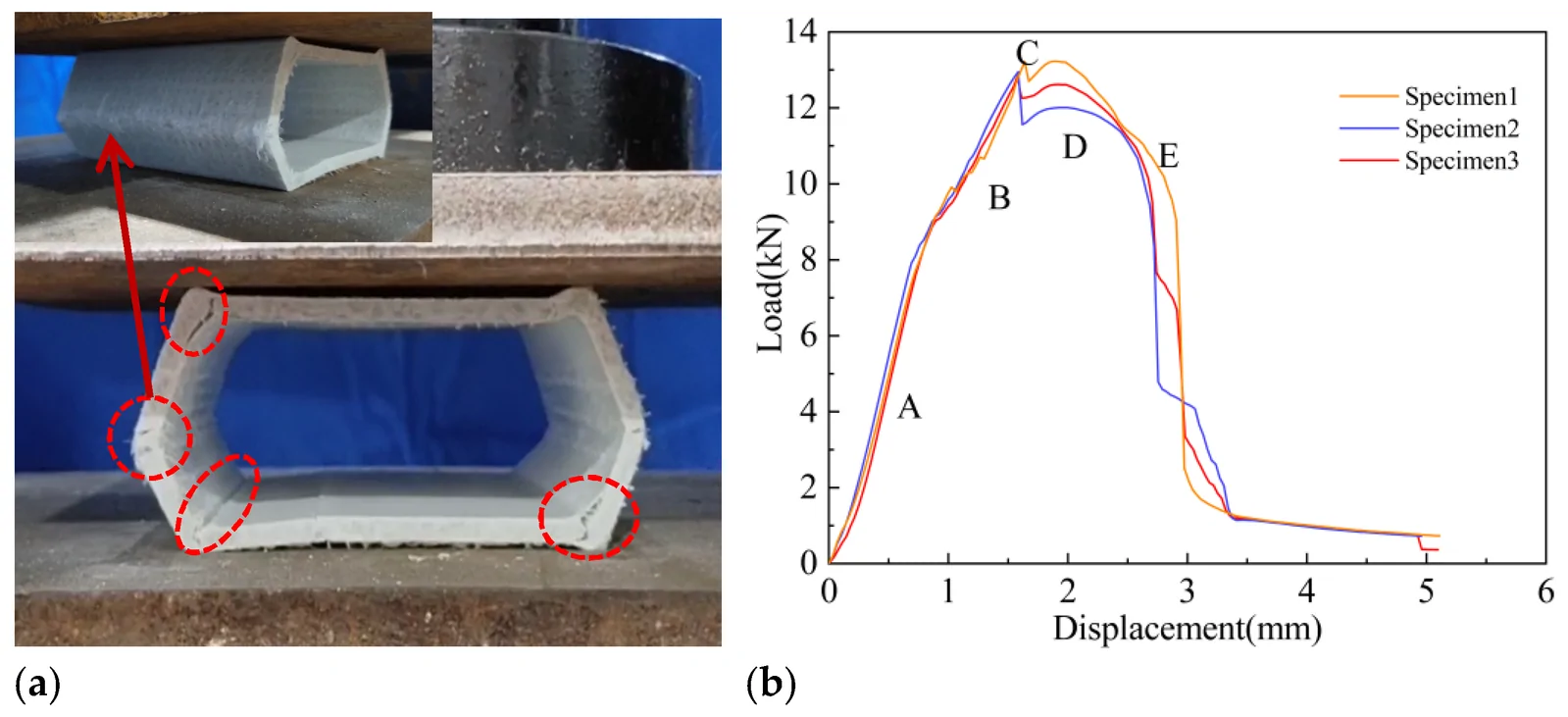
Failure mode and load–displacement responses of the CPH-S specimens. (**a**) Failure mode, with dashed red circles highlighting representative damaged regions. (**b**) Load–displacement responses. Labels A–E indicate characteristic stages of the response, including initial loading, progressive web deformation, peak load, post-peak damage development, and the onset of rapid capacity loss, respectively.

Figure 14 shows the failure mode and load–displacement responses of the CPH-S specimens. Load initially increased approximately linearly with displacement. The two webs then deformed outwards, intermittent fibre-fracture sounds were heard, and the tangent stiffness decreased. A crack initiated on the inner surface of one wall and propagated through its thickness, causing a small load reduction. Further compression produced delamination within the inner plies of the top and bottom walls and increasingly pronounced flexural deformation of both webs. Finally, a longitudinal through-thickness crack formed near the mid-height of a web, and the delamination propagated towards the outer plies, resulting in rapid capacity loss. The three specimens reached peak loads of 13.23, 12.96 and 12.82 kN at displacements of 1.64, 1.59 and 1.58 mm, respectively.

**Figure 15:**
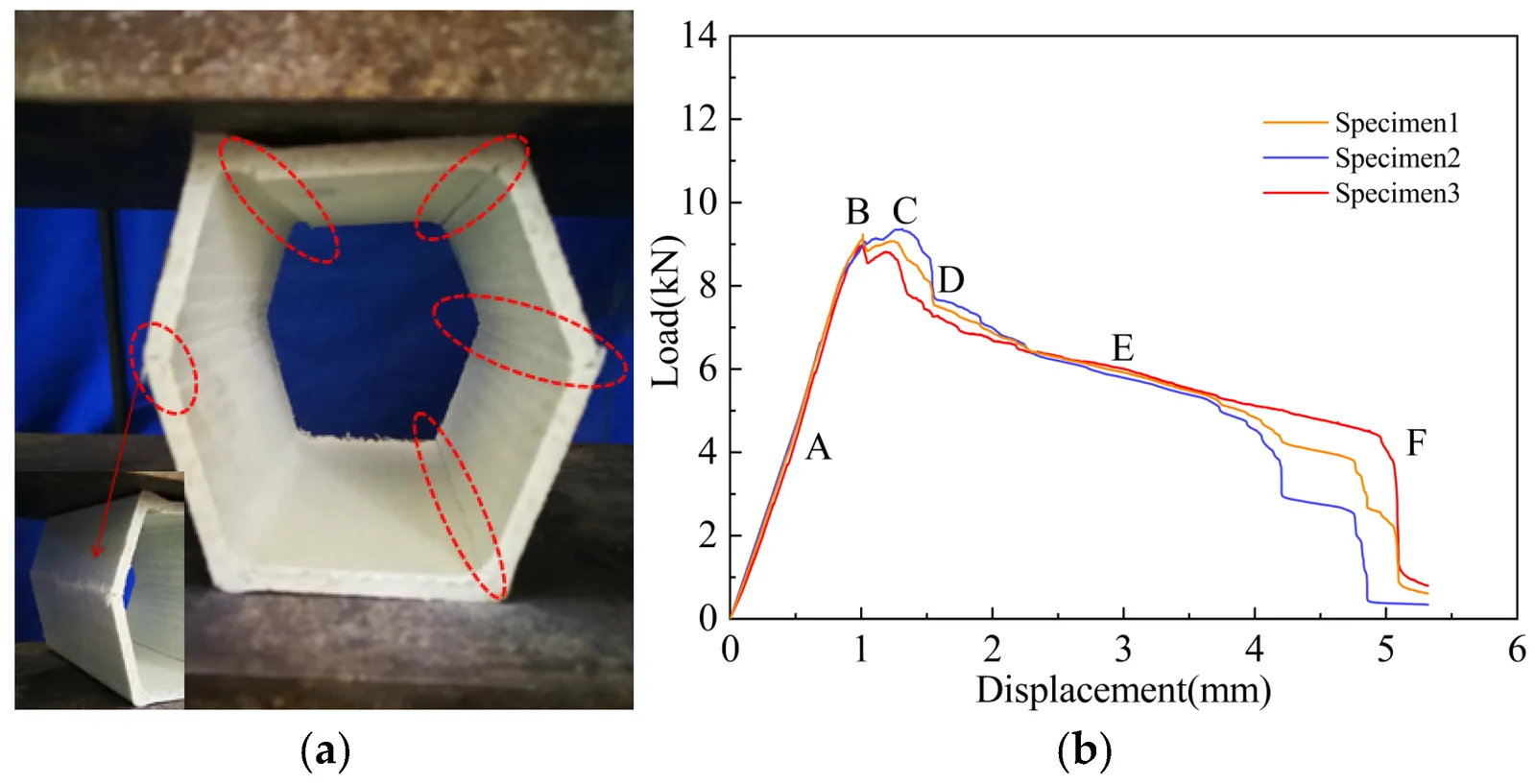
Failure mode and load–displacement responses of the CPV-S specimens. (**a**) Failure mode. (**b**) Load–displacement responses. Labels A–F denote characteristic stages of the response, including initial loading, onset of local web deformation and fibre fracture, peak load, post-peak cracking, progressive deformation, and final rapid capacity loss, respectively.

Figure 15 presents the load–displacement responses and failure mode of the CPV-S specimens. The initial response was approximately linear. Progressive outward web deformation was followed by fibre fracture at the inner surface of the upper wall, formation of a through-thickness crack and a small load reduction. Additional through-thickness cracks then developed in the upper and lower walls. During the post-peak stage, flexural deformation of both webs increased, and longitudinal through-thickness cracks formed near web mid-height, ultimately causing rapid loss of capacity. Peak loads of 8.96, 9.07 and 9.23 kN occurred at displacements of 0.99, 1.03 and 1.02 mm, respectively.

Table 3 summarises the flatwise compressive strength and chord modulus. The CPH-S group had mean values of 13.33 and 48.73 MPa, respectively, compared with 9.31 and 39.90 MPa for CPV-S. Relative to the H orientation, the V orientation produced taller, narrower webs, larger web slenderness and smaller projected loaded width. These geometric changes increased susceptibility to outward web deformation and local instability under transverse compression.

#### 3.1.6. Effect of Cross-Sectional Orientation

Table 4 compares representative H- and V-oriented specimens under the three loading conditions. The effect of orientation depended strongly on the governing load state. Changing from H to V increased the section depth from 50 to 80 mm. The four-point bending peak load rose from 11.98 to 13.10 kN (9.35%), and the short-span three-point bending peak load rose from 8.98 to 11.32 kN (26.06%). In contrast, the mean flatwise compression peak load decreased from 13.00 to 9.09 kN (30.12%).

In four-point and short-span bending, the deeper V-oriented section increased global flexural rigidity and may have increased the effective depth participating in shear transfer. The accompanying increase in web slenderness, however, promoted local instability and limited the capacity gain. Under flatwise compression, projected loaded width and web stability became dominant, so the same reorientation reduced capacity. Profile orientation should therefore be selected with reference to the expected load-transfer mechanism rather than section depth alone. These orientation-dependent failure trends are consistent with previous studies of pultruded GFRP sections, which identified web crippling, local buckling and cracking near wall–web junctions as important transverse failure modes and showed their sensitivity to fibre lay-up, bearing configuration and section geometry [13,14,15,16,17,18,19,20]. The present tests further show that rotating the same rectangular hollow profile can have opposite effects under different loading conditions. Increasing the section depth in the V orientation was beneficial under four-point and short-span bending, whereas the accompanying increase in web slenderness reduced resistance under flatwise compression.

### 3.2. Mechanical Response of Integrated Pultruded-Profile-Core Sandwich Panels

#### 3.2.1. Failure Evolution Under Four-Point Bending

**Figure 16:**
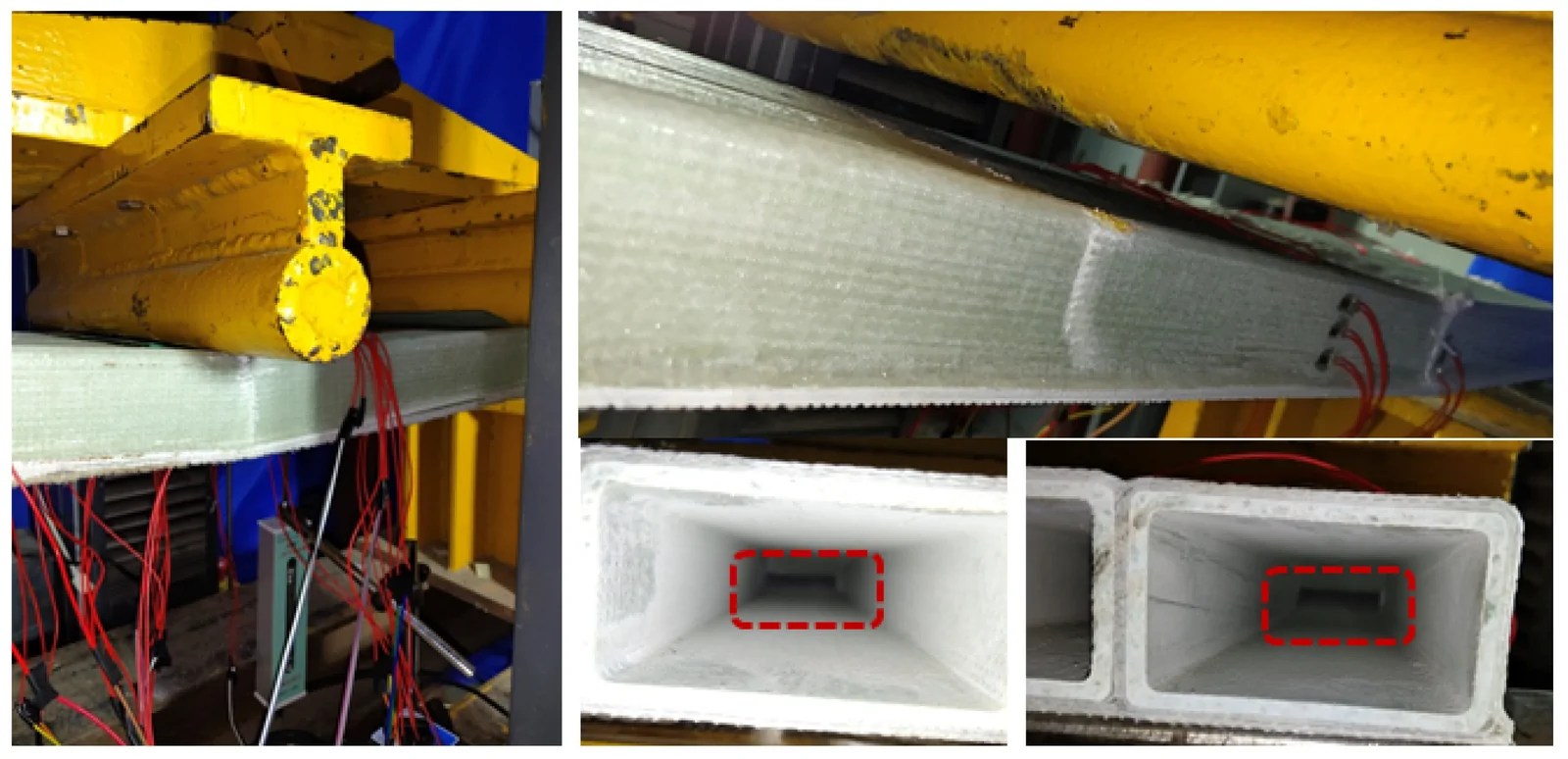
Failure modes of the FPH sandwich panel. Dashed red rectangles highlight representative local damage regions in the internal pultruded-profile core.

Figure 16 shows the failure mode of FPH. The initial load–displacement response was approximately linear. Faint damage-related sounds were first detected at 15.32 kN and became more frequent above 63.68 kN, indicating progressive local damage in the internal profiles and external face sheet near the loading points.

The panel reached a peak load of 73.60 kN. At peak load, shear failure developed in the profile-core webs beneath the loading points, while the top face sheet underwent local crushing and through-thickness cracking. Cracks then propagated down the side wrapping laminate towards the bottom face sheet, and the capacity fell rapidly to approximately 32.77 kN. The observed sequence suggests that core-web shear damage first disrupted load transfer, followed by local failure of the top face sheet under combined contact and flexural compression.

**Figure 17:**
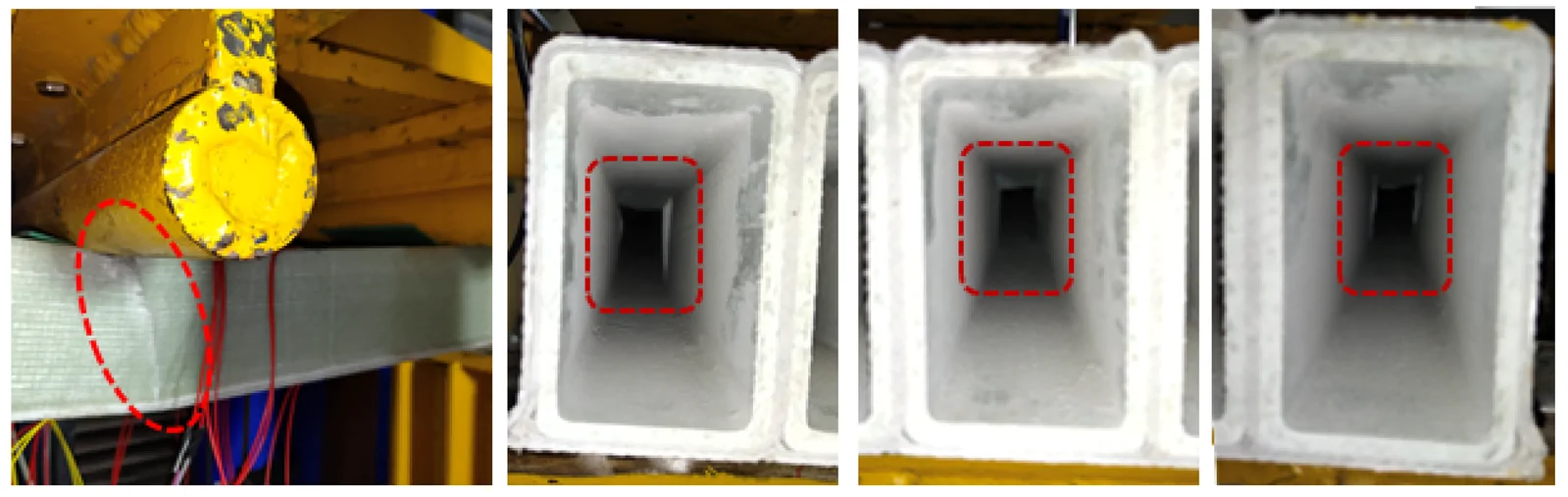
Failure modes of the FPV sandwich panel. Dashed red outlines highlight representative local damage regions.

FPV also retained an approximately linear response before peak load. Following successive fibre-fracture sounds, the panel reached 90.34 kN. Shear damage in the profile-core webs beneath the loading points caused an initial load reduction, after which the local crushing of the top face sheet reduced the capacity rapidly to approximately 63.57 kN. Cracking subsequently extended from the loaded region towards the outer core web and the bottom face sheet. The capacity decreased to approximately 44.71 kN and then approached a relatively stable residual level. The observed failure modes of FPV are shown in Figure 17.

In both FPH and FPV, failure resulted from interaction between local shear damage in the internal profile core and failure of the top face sheet near the loading points. Compared with the individual profiles, the continuous face sheets and wrapping laminates restrained local profile deformation and delayed its development into global panel failure. The substantial residual capacity after local core damage is consistent with continued load transfer through the external laminates and adjacent profiles, although the load carried by individual profiles was not measured directly.

#### 3.2.2. Load–Displacement Response and Effect of Core Orientation

**Figure 18:**
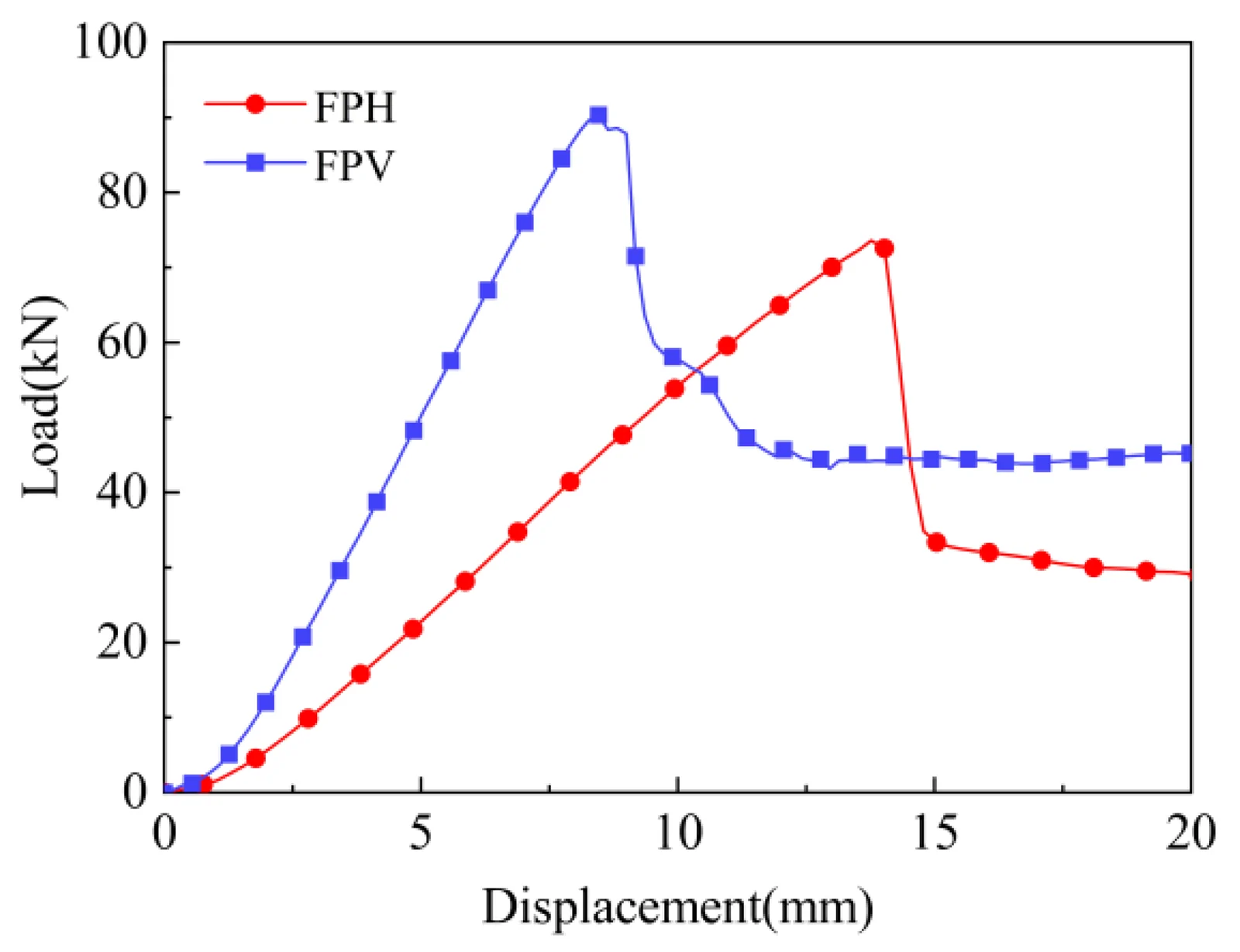
Load–displacement responses of the FPH and FPV sandwich panels.

Figure 18 compares the load–displacement responses of FPH and FPV. FPH reached 73.60 kN at 13.77 mm, whereas FPV reached 90.34 kN at 8.46 mm. Changing from the H to the V core orientation increased peak load by 22.74% and reduced displacement at peak load by 38.56%.

The V orientation increased core depth from 50 to 80 mm and therefore increased the separation of the face sheets and the global flexural rigidity of the panel. This change is consistent with the higher peak load and smaller displacement at peak of FPV. Nevertheless, failure in both panels remained concentrated beneath the loading points; the deeper section did not remove the susceptibility of the core webs to local shear damage or of the top face sheet to contact crushing.

#### 3.2.3. Strain Development and Sectional Response

**Figure 19:**
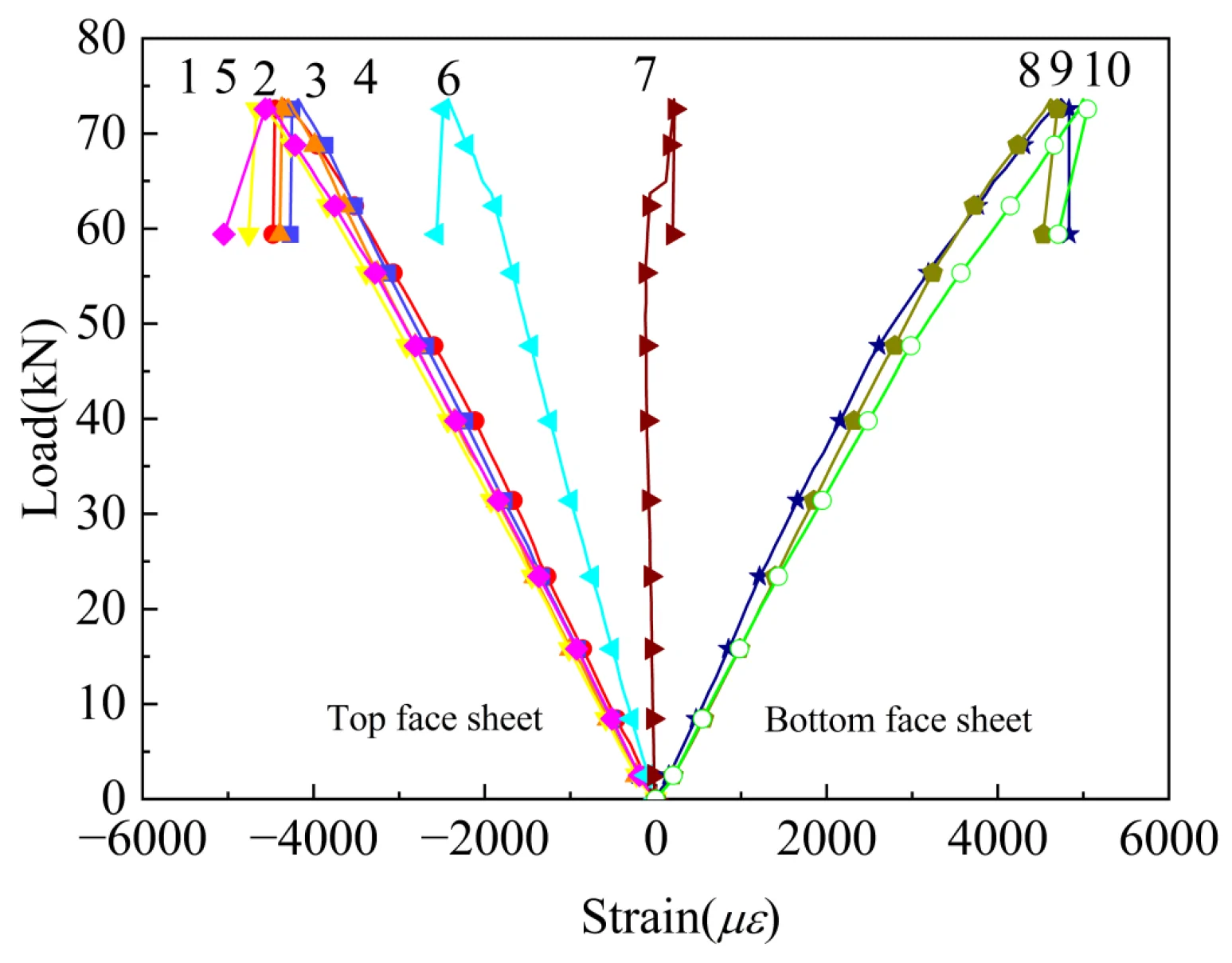
Load–strain responses of the FPH sandwich panel. Numbers indicate the strain-gauge locations shown in the instrumentation scheme.

**Figure 20:**
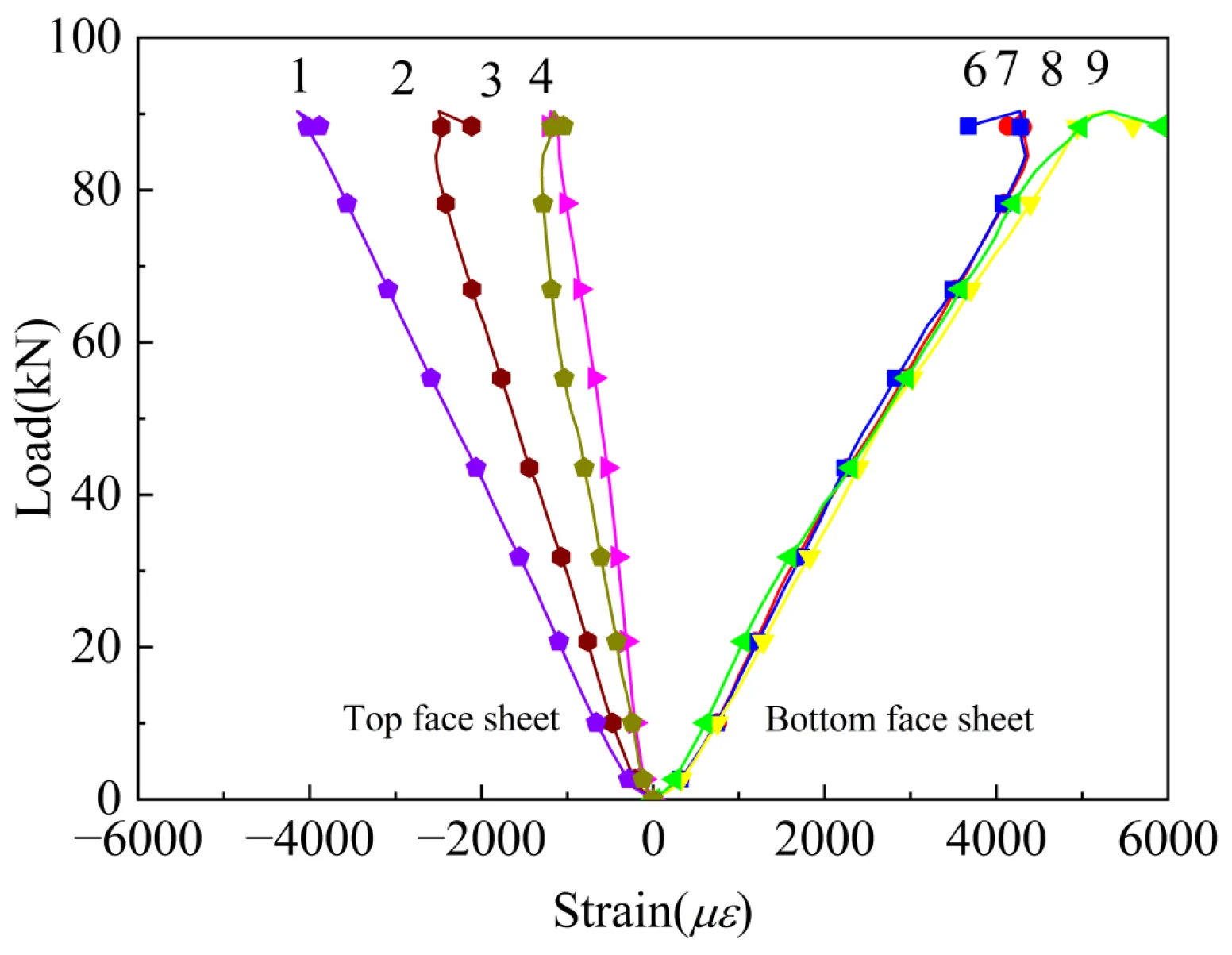
Load–strain responses of the FPV sandwich panel. Numbers indicate the strain-gauge locations shown in the instrumentation scheme.

Figure 19 and Figure 20 show the load–strain responses of FPH and FPV, respectively. Strains at the instrumented locations generally varied approximately linearly with load before peak capacity, followed by abrupt changes as core shear damage and local face-sheet failure developed. In FPH, the maximum tensile strain in the bottom face sheet was approximately 0.512%, well below the measured ultimate tensile strain of 1.54%. The maximum compressive strain in the top face sheet reached approximately 0.505%, close to the measured ultimate compressive strain of 0.52%.

Together with the observed damage sequence, these measurements indicate that shear damage developed first in the pultruded-profile core and was followed by the local crushing and cracking of the top face sheet beneath the loading points. Because the bottom face-sheet strain remained well below its tensile limit, tensile rupture of the bottom face sheet did not govern peak capacity.

**Figure 21:**
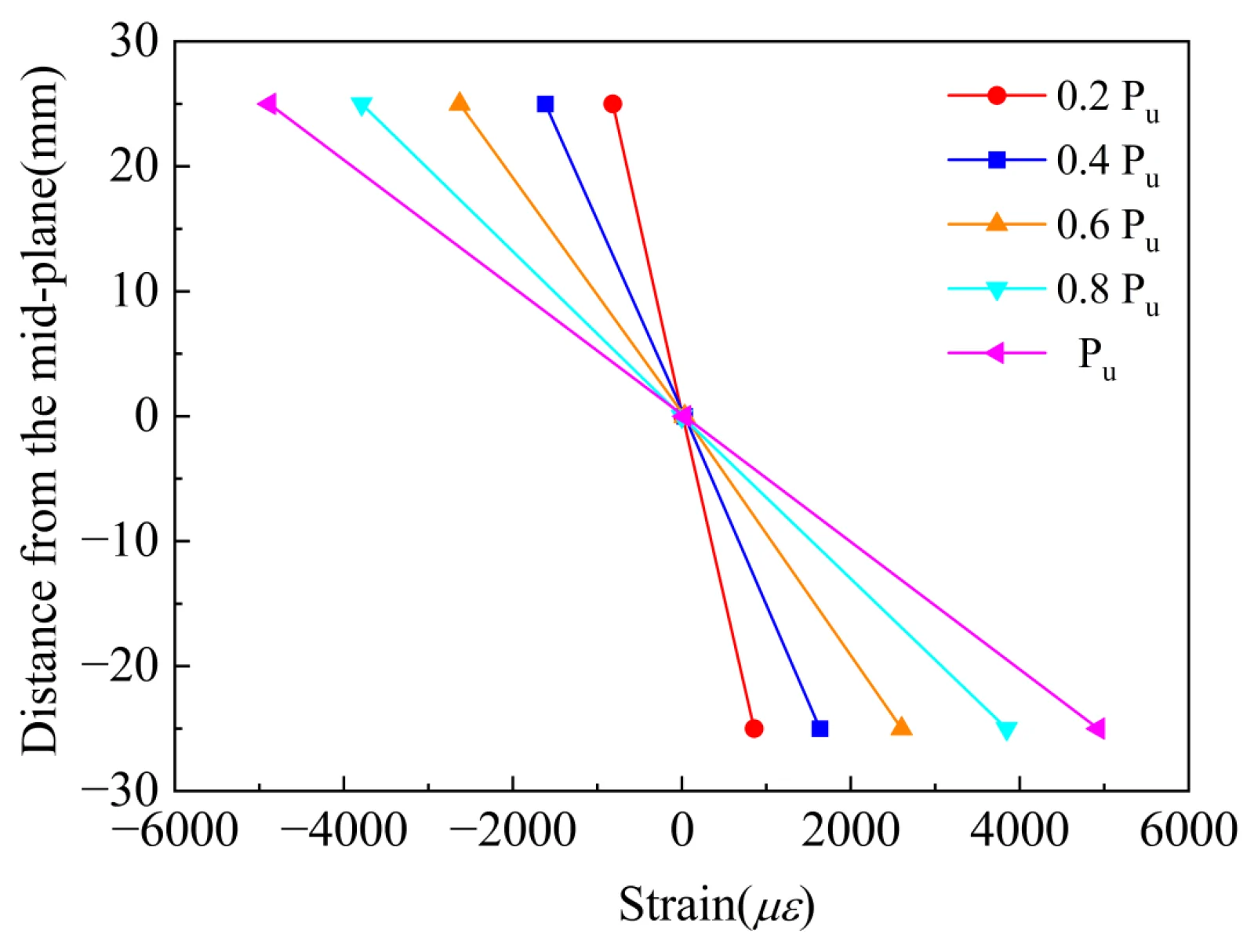
Longitudinal strain distributions through the depth at midspan for the FPH sandwich panel.

**Figure 22:**
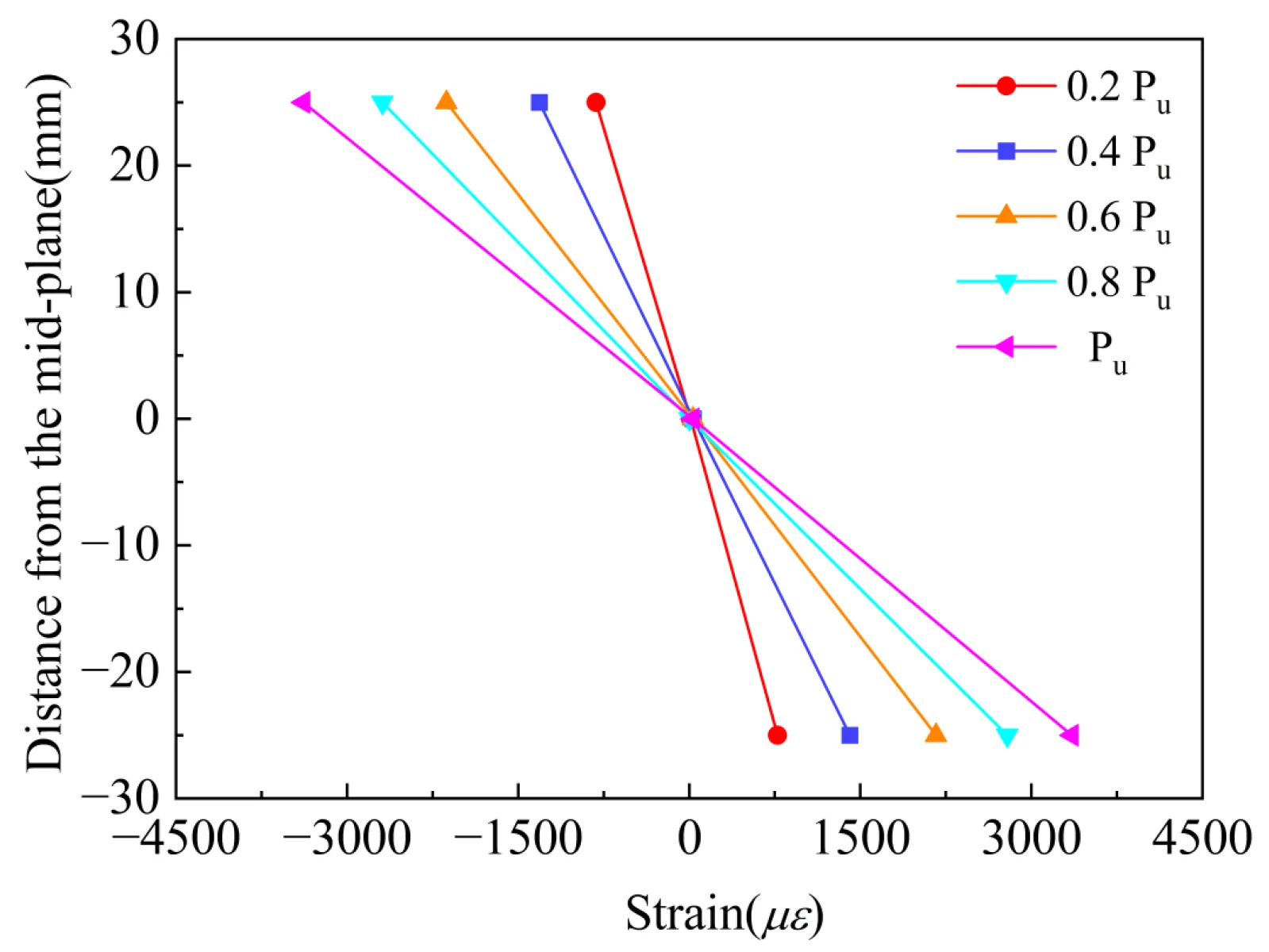
Longitudinal strain distributions through the depth at midspan for the FPV sandwich panel.

Figure 21 and Figure 22 show the midspan longitudinal strain distributions of FPH and FPV at 0.2 Pu, 0.4 Pu, 0.6 Pu, 0.8 Pu and Pu. In both panels, longitudinal strain varied approximately linearly with distance from the mid-plane. This through-depth distribution indicates compatible sectional deformation up to peak load and joint participation of the face sheets, wrapping laminates and internal profiles in resisting flexure at midspan.

#### 3.2.4. Short-Span Three-Point Bending Response and Failure Mechanisms

SPH showed an approximately linear initial response. At 58.27 kN, the tangent stiffness began to decrease as the upper wall of the profile beneath the loading point deflected downwards and developed local inward buckling. The panel reached a peak load of 81.32 kN, after which shear failure in the core web beneath the loading point reduced the capacity to 73.13 kN.

**Figure 23:**
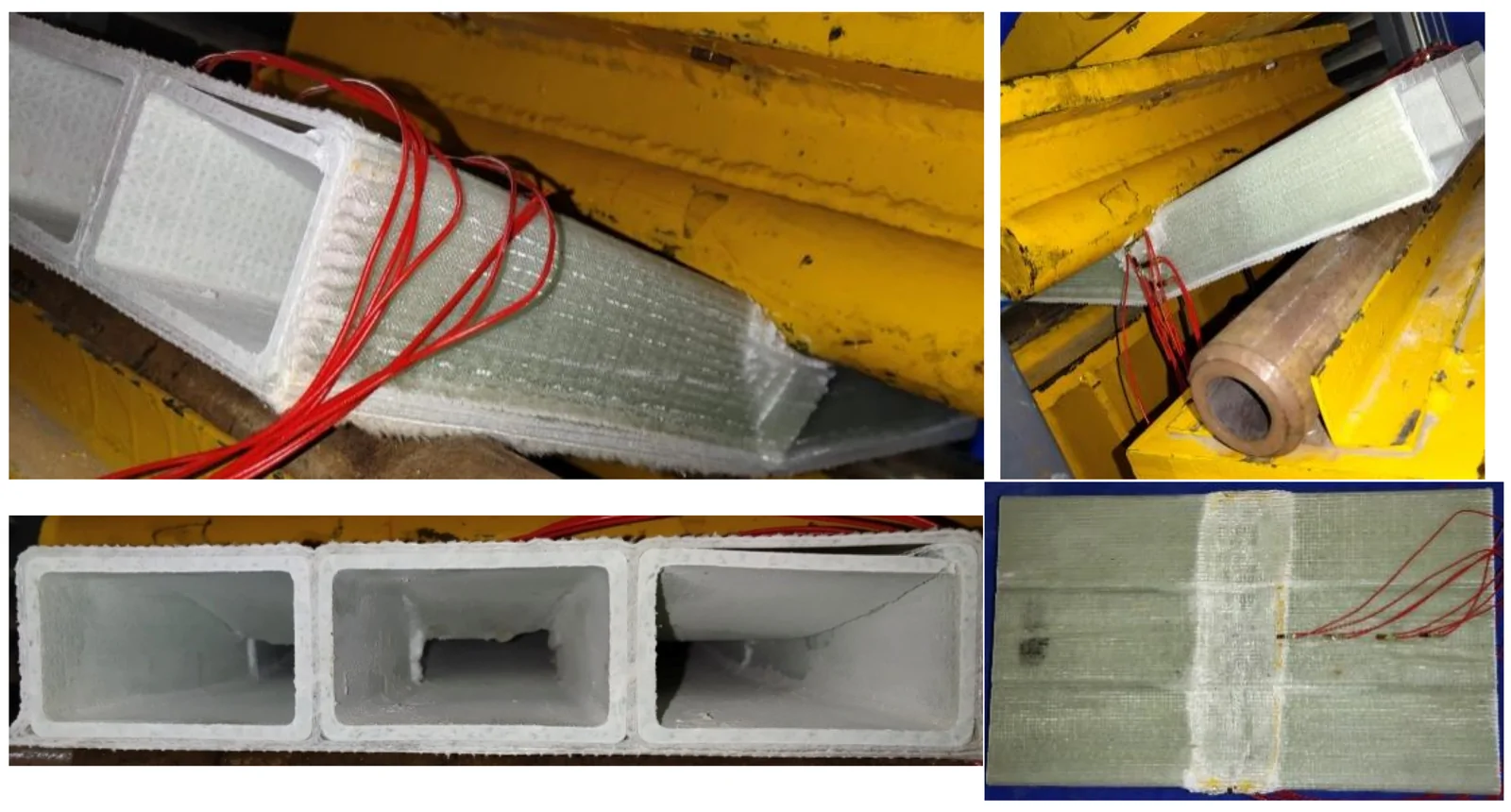
Failure modes of the SPH sandwich panel.

Web cracking then propagated downwards from the loading point. Local indentation developed in the top face sheet, accompanied by debonding between the outermost pultruded profile and the surrounding wrapping laminate. Accumulating damage caused a fluctuating reduction in capacity, although the panel retained substantial residual resistance. Figure 23 shows the final damage pattern.

SPV likewise exhibited an approximately linear initial response. Shear failure developed in the core web beneath the loading point at 82.90 kN and reduced the capacity to 75.66 kN. After a brief recovery, the load fell to 69.44 kN as the outer web deformed outwards and developed vertical cracking.

**Figure 24:**
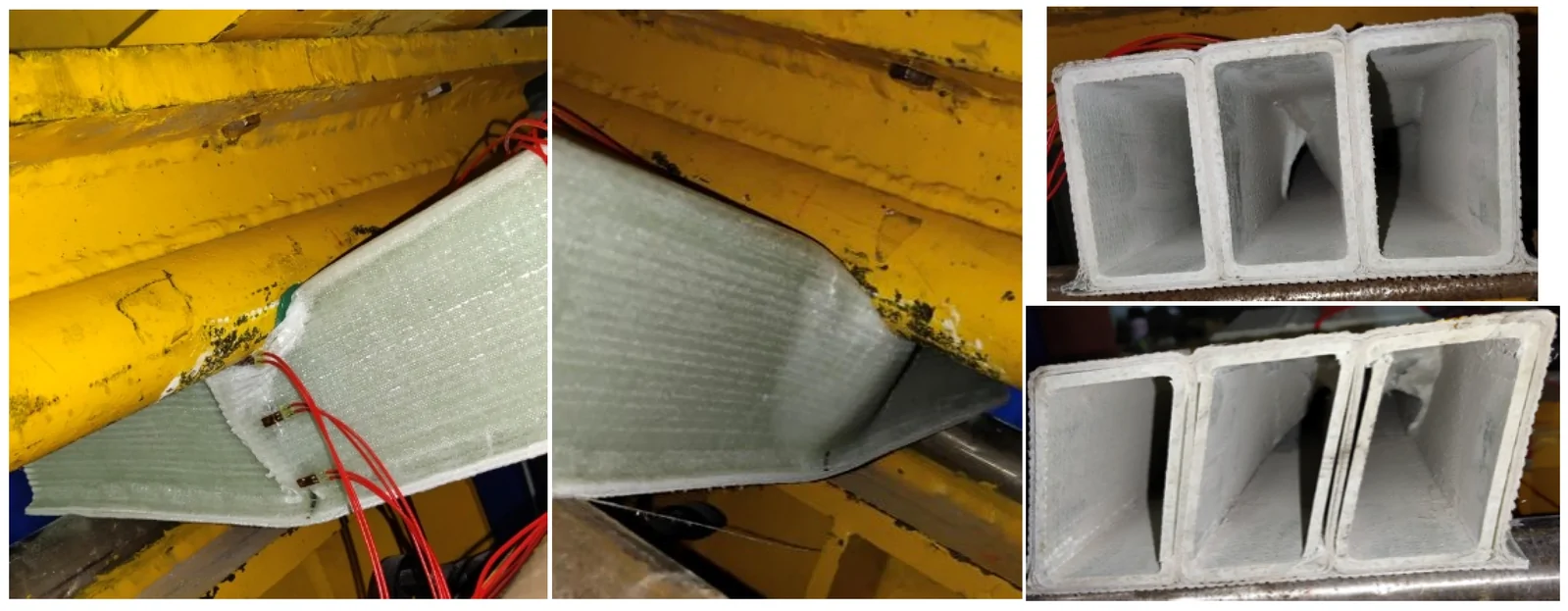
Failure modes of the SPV sandwich panel.

Damage subsequently spread through the internal core and produced asymmetric deformation towards one side. Delamination developed within the wrapping laminates, together with debonding at the profile–wrapping interfaces. Capacity gradually declined to approximately 59.37 kN and then remained comparatively stable. The observed failure mode is shown in Figure 24.

**Figure 25:**
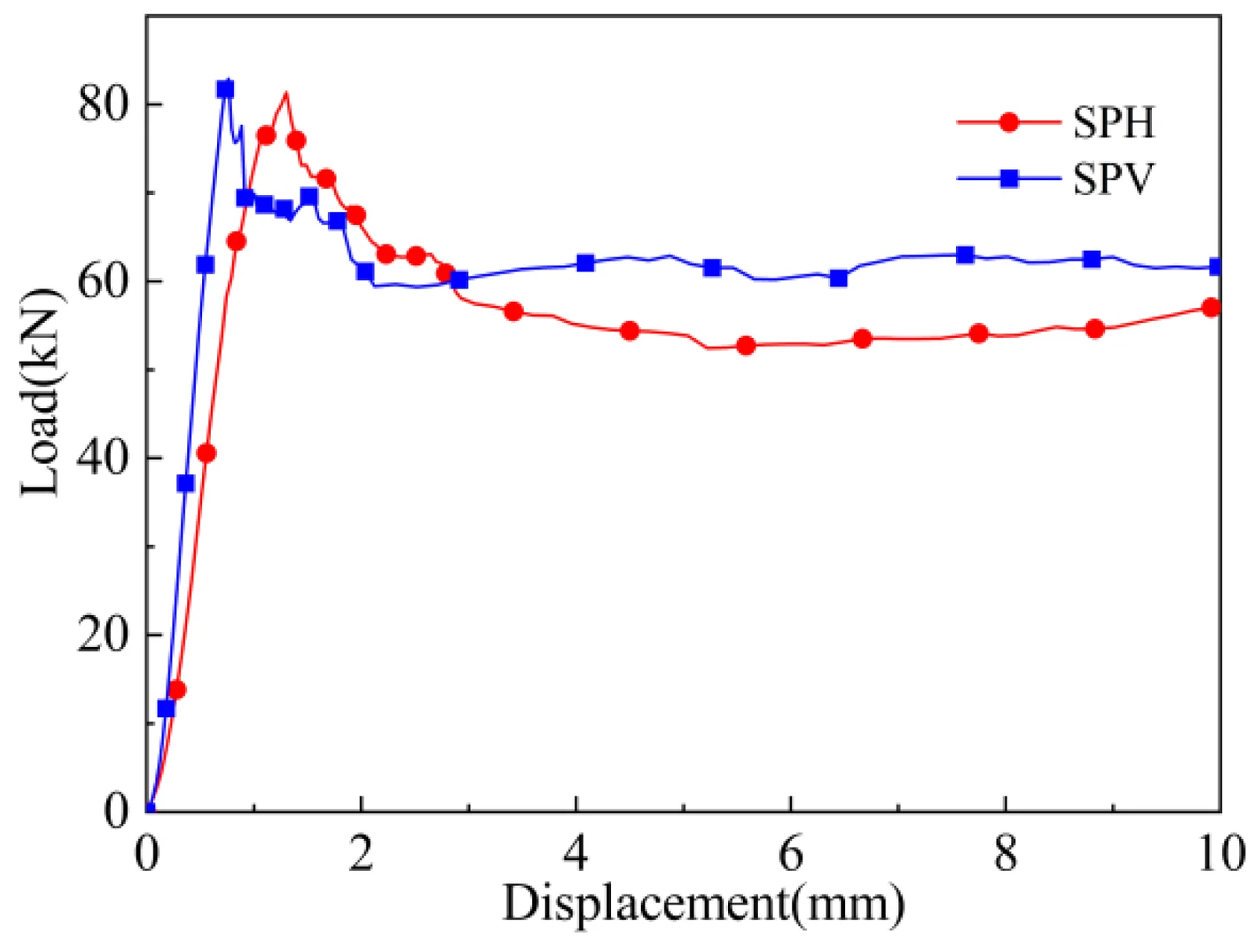
Load–displacement responses of the SPH and SPV sandwich panels.

As shown in Figure 25, SPH and SPV reached peak loads of 81.32 and 82.90 kN, a difference of only 1.58 kN. Their displacements at peak load were 1.30 and 0.76 mm, respectively; the value for SPV was 41.54% lower.

#### 3.2.5. Flatwise Compression Response and Failure Mechanisms

**Figure 26:**
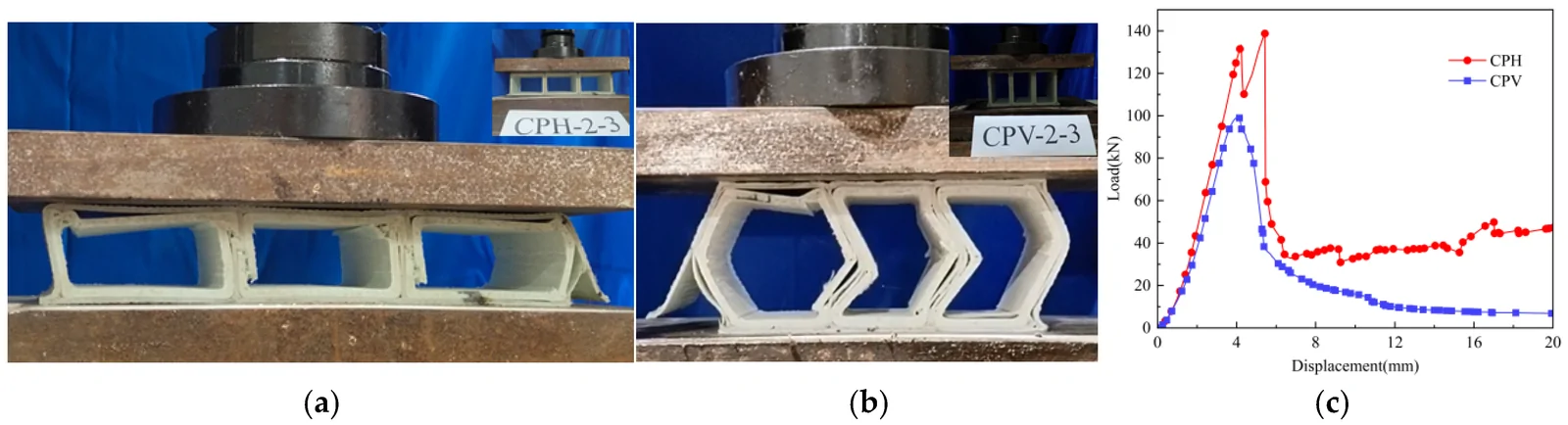
Failure modes and load–displacement responses of the CPH and CPV sandwich panels under flatwise compression. (**a**) Failure mode of CPH. (**b**) Failure mode of CPV. (**c**) Load–displacement responses.

Figure 26 presents the failure modes and load–displacement responses of CPH and CPV under flatwise compression. Both panels showed an approximately linear initial response, but core orientation had a pronounced effect on peak capacity and post-peak behaviour.

CPH reached an initial local peak of 133.32 kN at 4.24 mm. Flexural deformation of the internal wrapping laminates, the diagonal cracking of the pultruded-profile webs under combined compression and transverse shear, and local debonding between an outer wrapping laminate and the adjacent profile web then reduced the load to approximately 110.17 kN. Because the damage remained localised, the panel recovered and reached a final peak of 138.77 kN at 5.43 mm. Beyond this peak, damage propagated rapidly through the internal laminates and along profile–wrapping interfaces, producing a pronounced capacity reduction followed by a fluctuating residual-load stage.

CPV reached a peak load of 99.58 kN at 4.04 mm. Its taller profile webs progressively deformed outwards, accompanied by local debonding between the internal wrapping laminates and profile webs. Further compression extended debonding between the external composite layers and the profile core. Capacity then decreased rapidly after peak load, giving CPV a more brittle post-peak response than CPH.

The final peak loads of CPH and CPV were 138.77 and 99.58 kN, respectively; CPV was 28.24% lower. Although the V orientation increased core depth, it also reduced the projected loaded width and increased the slenderness of the profile webs. The resulting susceptibility to outward deformation and local instability made the V-oriented core unfavourable under flatwise compression.

To account for the small differences in specimen mass between the H- and V-oriented panels, the peak loads were also normalised by specimen mass. The mass-normalised peak loads were 7.67 and 9.77 kN/kg for FPH and FPV, 21.12 and 22.11 kN/kg for SPH and SPV, and 154.19 and 110.64 kN/kg for CPH and CPV, respectively. The corresponding H-to-V changes were +27.4%, +4.7% and −28.2%. Thus, normalisation by specimen mass did not alter the overall orientation-dependent trends observed from the absolute peak loads.

### 3.3. Comparison Between Component-Wise and Integrated-Panel Capacities

#### 3.3.1. Component-Wise Superposition Baseline and Measured Panel Capacities

Composite action was evaluated by comparing the measured peak load of each H-oriented integrated panel with a component-wise superposition baseline. The comparison was restricted to the H-oriented configuration because hollow FRP reference panels were available only for this orientation; corresponding V-oriented hollow reference panels were not tested. The baseline was the sum of the independently measured peak loads of three individual H-oriented profiles and the corresponding hollow FRP reference panel. Table 5 reports the component capacities, baseline values and measured integrated-panel capacities.

The component-wise baselines for four-point bending, short-span three-point bending and flatwise compression were 63.58, 59.05 and 77.77 kN, respectively. The corresponding measured panel capacities were 73.60, 81.32 and 138.77 kN, exceeding the baselines by 15.76%, 37.71% and 78.44%. These differences constitute apparent capacity enhancements relative to independently tested components and provide experimental evidence of composite action. The largest enhancement occurred under flatwise compression. Because the integrated-panel and hollow reference configurations were represented by single specimens, the calculated enhancement percentages are descriptive values for the tested configurations rather than statistical estimates. For flatwise compression, the final peak load of 138.77 kN gives an enhancement of 78.44%, whereas use of the initial local peak of 133.32 kN gives 71.43%. This comparison indicates that the magnitude of the calculated enhancement is sensitive to the definition of peak load.

#### 3.3.2. Effect of Integrated Fabrication on Load Transfer and Damage Interaction

The measured integrated-panel capacities exceeded the component-wise baselines, indicating that panel performance was not reproduced by simple addition of the independently tested components. Vacuum infusion connected the face sheets, wrapping laminates and pultruded profiles into a continuous load-transfer system. The external laminates carried load directly, restrained outward web deformation and limited opening at profile–wrapping interfaces. After local core damage, the retained panel capacity was consistent with redistribution of load within the integrated section; however, load transfer between individual profiles was not measured directly.

Under four-point and short-span three-point bending, severe damage still initiated primarily in the profile webs beneath the loading points. Transverse shear resistance and local web stability therefore remained governing weaknesses. The continuous external laminates nevertheless slowed the progression from local damage to global failure and allowed the panels to retain appreciable post-damage capacity.

The apparent enhancement under flatwise compression was 78.44%, substantially greater than under the two bending configurations. In this load state, the wrapping laminates simultaneously restrained outward deformation and interfacial opening across several profile webs. The integrated architecture also preserved alternative load paths after local web instability developed. These observations are consistent with a stronger manifestation of composite action under flatwise compression, although the enhancement index remains specific to the component-wise baseline adopted here. Previous studies on pultruded-profile-core sandwich systems have similarly reported the importance of core shear damage and interfacial debonding under short-span loading and the influence of multiaxial wrapping on flexural failure and post-peak resistance [37,38]. The present study complements these observations by comparing the integrated panels with separately tested components under three loading conditions. The different enhancement values obtained under four-point bending, short-span three-point bending and flatwise compression indicate that the apparent composite action depends on the loading condition and structural configuration rather than representing a constant material property.

## 4. Conclusions

Rectangular hollow pultruded GFRP profiles and integrated sandwich panels manufactured from three identical profiles were tested under four-point bending, short-span three-point bending and flatwise compression. The principal findings are as follows:

1. Profile orientation affected the individual specimens in a load-state-dependent manner. Changing from H to V increased peak load by 9.35% in four-point bending and by 26.06% in short-span three-point bending but reduced the flatwise compression peak load by 30.12%. Mean flatwise compressive strength and chord modulus decreased from 13.33 and 48.73 MPa for CPH-S to 9.31 and 39.90 MPa for CPV-S. The deeper V-oriented section was advantageous in bending-related loading but less stable under transverse compression.
2. Failure of the individual profiles in four-point and short-span bending was controlled by local damage beneath the loading points, including web buckling, web shear damage and longitudinal splitting at wall–web junctions. Midspan strains remained approximately linear through the depth before peak load, indicating compatible global bending. Peak capacity was nevertheless limited by local bearing and the transverse stability and damage resistance of the loaded webs.
3. Core orientation also produced different effects in the integrated panels. Changing from FPH to FPV increased peak load from 73.60 to 90.34 kN (22.74%) and reduced displacement at peak by 38.56%. SPH and SPV reached similar peak loads of 81.32 and 82.90 kN, although the displacement at peak of SPV was 41.54% lower. Under flatwise compression, CPV reached 99.58 kN, 28.24% below the 138.77 kN of CPH. A deeper core therefore improved flexural stiffness but increased the vulnerability of taller profile webs to outward deformation and local instability under flatwise compression.
4. The integrated panels exceeded the component-wise superposition baselines by 15.76%, 37.71% and 78.44% under four-point bending, short-span three-point bending and flatwise compression, respectively. The apparent enhancements were associated with direct load participation of the face sheets and wrapping laminates, restraint of local profile deformation and redistribution of load among adjacent profiles. Composite action was the most pronounced under flatwise compression, but the measured enhancement remained dependent on the specific baseline and test configurations used.

## Figures and Tables

**Table 1:** Design parameters of the individual-pultruded-profile specimens.

Specimen	Test Type	Profile Orientation	Cross-Section (mm × mm)	Nominal Wall Thickness (mm)	Specimen Length (mm)	Span (mm)
FPH-S	Four-point bending	H	80 × 50	4	1000	800
FPV-S	Four-point bending	V	50 × 80	4	1000	800
SPH-S	Short-span three-point bending	H	80 × 50	4	400	300
SPV-S	Short-span three-point bending	V	50 × 80	4	400	300
CPH-S	Flatwise compression	H	80 × 50	4	100	—
CPV-S	Flatwise compression	V	50 × 80	4	100	—

**Table 2:** Design parameters of the integrated pultruded-profile-core sandwich panels.

Specimen	Test Type	Core Orientation	Wrapping Lay-Up	Face-Sheet Lay-Up	Specimen Length (mm)	Span (mm)
FPH	Four-point bending	H	[(±45°)_2_]	[(0°,90°)/(±45°)/(0°,90°)]	1000	800
FPV	Four-point bending	V			1000	800
SPH	Short-span three-point bending	H			400	300
SPV	Short-span three-point bending	V			400	300
CPH	Flatwise compression	H			100	—
CPV	Flatwise compression	V			100	—

**Table 3:** Flatwise compressive strength and chord modulus of the individual pultruded profiles.

Specimen		Strength (MPa)	Mean (MPa)	SD	CoV (%)	Chord Modulus (MPa)	Mean (MPa)	SD	CoV (%)
CPH-S	1	13.56	13.33	0.18	1.31	50.53	48.73	1.27	2.61
	2	13.28				47.75			
	3	13.14				47.92			
CPV-S	1	9.18	9.31	0.12	1.24	39.51	39.90	0.33	0.83
	2	9.29				39.86			
	3	9.46				40.32			

**Table 4:** Comparison of peak loads between H- and V-oriented individual pultruded profiles.

H-Oriented Specimen	Depth/Width Ratio	Peak Load (kN)	V-Oriented Specimen	Depth/Width Ratio	Peak Load (kN)	Change from H to V (%)
CPH-S	0.625	13.00 ± 0.21	CPV-S	1.60	9.09 ± 0.14	−30.12
FPH-S		11.98	FPV-S		13.10	9.35
SPH-S		8.98	SPV-S		11.32	26.06

Note: The four-point bending values are group mean peak loads (*n* = 3). The flatwise compression values are reported as means ± SD (*n* = 3). The short-span three-point bending values correspond to the illustrative specimens shown in the load–displacement curves.

**Table 5:** Comparison of component-wise baseline and integrated-panel peak loads for the H-oriented configuration.

Loading Condition	Single-Profile Peak Load (kN)	Three Profiles (kN)	Hollow FRP Reference Panel (kN)	Component-Wise Baseline (kN)	Integrated Panel (kN)	Enhancement (%)
Four-point bending	11.98	35.94	27.64	63.58	73.60	15.76
Short-span three-point bending	8.98	26.94	32.11	59.05	81.32	37.71
Flatwise compression	12.96	38.88	38.89	77.77	138.77	78.44

Note: For flatwise compression, the final peak load of 138.77 kN was used in the primary comparison. If the initial local peak of 133.32 kN is adopted instead, the corresponding apparent enhancement decreases from 78.44% to approximately 71.43%.

## Data Availability

The original contributions presented in this study are included in the article. Further inquiries can be directed to the corresponding author.
